# Aspectual reduplication in Sign Language of the Netherlands: reconsidering phonological constraints and aspectual distinctions

**DOI:** 10.1515/ling-2022-0076

**Published:** 2024-06-06

**Authors:** Cindy van Boven

**Affiliations:** University of Amsterdam Amsterdam, The Netherlands

**Keywords:** reduplication, aspect, phonological constraints, Sign Language of the Netherlands

## Abstract

This study investigates the use of predicate reduplication to express aspectual meaning in Sign Language of the Netherlands (NGT). The study focuses on three aspect types that have been found to be encoded by reduplication across sign languages – habitual, continuative, and iterative – and addresses potential phonological restrictions. Naturalistic corpus data and data elicited from six deaf NGT signers were taken into account. The results suggest that (i) predicate reduplication can express all three aspect types, but it is optional; (ii) reduplication expressing habitual and continuative aspect appears to be phonologically constrained; and (iii) such phonological constraints do not apply to iterative reduplication, whose form is different from the other two aspects, in that the reduplication cycles are separated by pauses. Since there is no formal difference between habituals and continuatives in the data, it is suggested that this semantic distinction may not be grammaticalized in the language, and that, possibly, the inflectional system of NGT instead more broadly distinguishes imperfective/perfective viewpoint. While this latter suggestion is in line with findings reported for many spoken languages, the results are different from what has previously been described for NGT as well as for other sign languages. Potential explanations for these differences can be found in both methodological and sociolinguistic factors.

## 1 Introduction

There is a long line of research on tense and aspect, investigating a vast variety of typologically diverse spoken languages. While tense has to do with time reference, aspect, which is the focus of the present study, relates to internal temporal structure (e.g., Binnick 2012a; Bybee 1985; Comrie 1976; Dahl 1985; Smith 1997). Both are traditionally considered grammatical categories of verbs: languages may reflect temporal and aspectual differences in the inflectional distinctions they make (compare, for instance, English present tense *walk* to past tense *walked*). Apart from that, periphrastic constructions may also play a role (e.g., the English progressive *he is walking*). Languages have been found to differ in the distinctions that are grammaticalized (see, e.g., Dahl and Velupillai [2013a] for further details).

As for sign languages, they have been shown to display striking similarities when it comes to the encoding of aspect and tense. Sign language verbs usually do not inflect for tense (but see, for instance, Zucchi [2009]). The most common strategy to express tense, i.e., to place an event on a timeline, is by means of adverbials, often using the signing space metaphorically (e.g., a backward movement in yesterday versus a forward movement in tomorrow; see Pfau et al. [2012] for an overview). In contrast, rich aspectual systems have been identified across sign languages (for an overview, see Pfau et al. [2012]). A common way of encoding aspectual distinctions is by modulating the verb’s movement component, be it by reduplicating the movement and/or by adapting its rate and rhythm (e.g., Klima and Bellugi 1979; Rathmann 2005). Interestingly, reduplication has also been found to commonly encode certain aspect types in spoken languages (e.g., Bybee 1985; Finegan 2014).

This study investigates reduplication of the predicate as a strategy to express grammatical aspect in Sign Language of the Netherlands (*Nederlandse Gebarentaal*, NGT). The focus will be on the habitual, continuative, and iterative – three aspect types that have been found to involve reduplication across sign languages (e.g., Cabeza Pereiro and Fernández Soneira 2004; Pfau et al. 2012; Rathmann 2005; Zeshan 2000). A thorough investigation of NGT is worthwhile: while there are previous studies on this topic, many questions remain unanswered. On the one hand, Hoiting and Slobin (2001) found that habitual and continuative aspect in NGT are expressed by different types of repeated elliptical modulation of the verb sign, and identified phonological constraints on this inflection. Oomen (2016), on the other hand, also found that both these aspect types in NGT are expressed by reduplication, but she did not observe elliptical modulation or phonological constraints. The present study is the first to comprehensively investigate all three aspect types in NGT, attempting to explain the diverging findings of previous studies, and taking into account potential (phonological) restrictions on aspectual reduplication. The investigation is based on two types of data: semi-spontaneous data extracted from the Corpus NGT, and data elicited from six deaf NGT signers by means of a novel elicitation task.

Section 2 provides some background on aspect in spoken and signed languages, including previous studies on NGT specifically, and concludes with the present study’s research questions. Section 3 details the method, addressing first the corpus, before detailing the elicitation task. This section also describes the data annotation and statistical analysis. Section 4 then provides an overview of the results, while Section 5 further discusses the phonological constraints on aspectual reduplication, takes a typological perspective on the aspectual distinctions NGT makes, and offers some methodological and sociolinguistic considerations. Section 6, finally, draws some conclusions.

## 2 Aspect in spoken and signed languages

### 2.1 General background

Previous work has shown that two types of aspect can be distinguished: lexical aspect and grammatical aspect. First, lexical aspect (also called “situation aspect” by Smith [1997]) refers to inherent properties of the verb, or, in the words of Filip (2012: 721), it is “a semantic category that concerns properties of eventualities (in the sense of Bach 1981) expressed by verbs”. These “properties” generally refer to an end or boundary that is present in the lexical structure of some classes of verbs, but not in others, known as the basic distinction between telic verbs, which have a clear endpoint or goal, and atelic verbs, which do not (Filip [2012]; and see Garey [1957] for the telic/atelic distinction). Two other fundamental concepts are change of state (i.e., whether there is a transition from one state to another) and temporal extent (i.e., whether the event is punctual or whether there is some temporal extent). Based on these three properties, verbs can be divided into different classes (for instance, states or processes) (Filip [2012], and references therein such as Bach [1986]; Comrie [1976]; Dowty [1979]; Vendler [1957]). The present study will not be concerned with lexical aspect, and solely focuses on grammatical aspect – although, of course, the two are closely related (see Binnick [2012a] for more discussion).1For sign languages, see also the Event Visibility Hypothesis (Wilbur 2003, 2008), which proposes that the phonological form of predicate signs reflects the semantics of the event structure, i.e., “predicate signs contain morphemes that reflect the event structure they represent. These morphemes have regular phonological forms by which they are recognized” (Wilbur 2008: 29). According to this hypothesis, the difference between telic and atelic verbs is thus marked at the morpho-phonological level of the predicate sign (see also, e.g., Krebs et al. [2021]; Krebs et al. [2023]; Strickland et al. [2015]).


Second, grammatical or verbal aspect (also called “viewpoint aspect” by Smith [1997]) is not an inherent lexical property of the verb, but can be described as “a sub-system belonging to the grammar of a particular language” (Binnick 2012b: 32). Comrie (1976: 3) defined it as “different ways of viewing the internal temporal constituency of a situation”. Taking English as an example, De Swart (2012: 753) compares *When Bill came to the office, Sarah left through the back door* to *When Bill came to the office, Sarah was leaving through the back door* – the progressive verb form in the second sentence changes the interpretation of the sentence and is part of the English inflectional system: it functions as a grammatical aspect marker. It is this type of aspect, i.e., grammatical aspect, that this study focuses on – specifically, continuative, habitual, and iterative aspect.

Within the domain of grammatical aspect, a fundamental distinction is that between perfective and imperfective aspect. Perfective viewpoints focus on the situation in its entirety, looking at the situation from outside, while imperfective viewpoints focus on part of a situation and its internal structure (see, e.g., Comrie 1976; Gvozdanović 2012; Smith 1997). While this distinction is grammaticalized in some languages (e.g., Russian and Spanish), this is not the case for all languages (e.g., English) (Comrie 1976; see also Gvozdanović 2012). Still, it has been noted that the distinction between these two viewpoints by means of different verbal inflectional forms is “fairly stable across languages” (Deo 2012: 161). Many languages have a single category that expresses the imperfective. Bybee (1985), for instance, noted that the distinction between perfective and imperfective is the most common inflectional aspectual distinction made in her sample of 50 languages. However, as we will see in Section 2.2, languages may also have further formalized distinctions, such as habitual and continuous aspect, which are usually assumed to be subtypes of the imperfective (Comrie [1976: 25]; see also, e.g., Carlson [2012] on habitual aspect; Mair [2012] on continuous aspect). The distinction between habitual/continuous is the second most common one in Bybee’s (1985) sample.

In the following, I first set the scene by describing previous studies on spoken languages, focusing on the three grammatical aspect types under investigation in the present research (Section 2.2). Then, I go into the two types of realization of grammatical aspect that have been identified for sign languages: modulating the verb sign (Section 2.3.1) and free functional elements (Section 2.3.2) (see also Pfau et al. [2012] for an overview). Finally, I turn to previous studies on NGT (Section 2.4), the language under investigation here.

### 2.2 Continuative, habitual, and iterative aspect in spoken languages

The present study focuses on continuative, habitual, and iterative aspect. Here, I define each aspect type, and briefly discuss how they are commonly expressed in different spoken languages.

According to Comrie (1976), the imperfective aspect can be further divided into habitual and continuous aspect, as mentioned above. In turn, the continuous is further divided into progressive and nonprogressive, where the progressive has been described as “the idea that an event is progressing dynamically over a time frame opened up by an utterance” (Mair 2012: 803). This time frame has been defined by Klein (1994) as the “topic time”, i.e., “the time span to which the speaker’s claim on this occasion is confined” (Klein 1994: 4). “Nonprogressive” includes situations in progress or nonprogressive states (see Mair [2012: 808], also for a critical evaluation of this distinction).

Here, I will generally focus on the continuous. Various languages have developed a grammatical category to express these semantics, which I will refer to as *continuative aspect* here.2Note that I will use “continuous” and “continuative” interchangeably, as both terms have been used in previous studies. An example is given in (1), which shows that continuative aspect is expressed by a prefix on the verb in Zapotec.

(1)

**Table:** 

***ku*** *-ka?a-beé*
cont-write-3sg.human
‘He is writing.’
(Zapotec; Bybee 1985: 142; Pickett 1953: 220)

Apart from marking on the verb, continuative aspect has also been found to be marked by periphrastic constructions or by adverbs or particles (see Mair 2012). For instance, in Russian the lexical adverb *dolgo* expresses ‘for a long time’ (Smith 1997: 251).

The other type of imperfective aspect concerns habituals, which “describe a situation that is characteristic of an extended period of time” (Comrie 1976: 28) – they are imperfective, because the situation is characteristic of the whole period. In the Zapotec example (2a), the *habitual aspect* is marked on the verb by the bound morpheme *ru* – comparing (2a) to (1) illustrates that habitual and continuous meaning are contrasted inflectionally in Zapotec, i.e., by means of the bound morphemes *ru-* and *ku-*, respectively (Bybee 1985). Cross-linguistically it is common to express habituality with a verbal affix, but it may also take the form of, for instance, an auxiliary or a periphrastic construction (Carlson 2012).

Comrie (1976) notes that habituality should be distinguished from iterativity (the repetition of a situation), because repetition alone is not sufficient to use an imperfective or habitual form. He points out that, if a situation is repeated a few times, each repetition can be viewed as a single instance, i.e., a situation on its own, which can be referred to with the perfective form. Further, according to Comrie (1976), a habitual form can refer to situations without iterativity, as in (2b), where English *used to* is considered a habitual marker (although note that Binnick [2005] actually argues against English “used to” as a habitual marker – I will not go into this discussion here). For further discussion of the difference between habitual and iterative aspect, see Bertinetto and Lenci (2012).

Bybee (1985) shows that some languages (15 out of 50 in her sample) employ a verbal marker to give iterative meaning to the verb, i.e., *iterative aspect*, as illustrated for Kiway in (2c), where the affix *-ti* expresses iterativity. Further, iterativity can be expressed by similar means as those mentioned above for the other two aspect types, for instance, adverbials (Bertinetto and Lenci 2012).

(2)

a.

**Table:** 

***ru*** *-ka?a-beé*
habit-write-3sg.human
‘He writes (regularly).’^3^
(Zapotec; adapted from Bybee 1985: 142; Pickett 1953: 220)

3 “Regularly” is not present in the translation in Bybee (1985: 142), but is added here to emphasize the habitual meaning.

b.

**Table:** 

*The Temple of Diana used to stand at Ephesus.*
(Comrie 1976: 27)

c.

**Table:** 

*arigi*	*arigi-* ***ti***
scratch	scratch-it
‘to scratch’	‘to scratch repeatedly’
(Kiway; Bybee 1985: 150; Ray 1933)

Finally, a grammatical aspect marker that commonly occurs not only in spoken languages, but also in sign languages, is reduplication. A spoken language example is given in (3).

(3)

**Table:** 

*mahuta*	*mahuta∼mahuta*
‘to sleep’	‘to sleep constantly’
(Motu; Finegan 2014: 48)	

The next section will illustrate the importance of reduplication for sign language aspect marking.

### 2.3 Aspect in sign languages

This section focuses on grammatical aspect in sign languages, which is mainly expressed by verbal inflection (specifically, reduplication) and free-standing markers. These will be discussed in turn in Sections 2.3.1 and 2.3.2, respectively. Section 2.4 outlines previous studies on aspect in NGT specifically, and finally, Section 2.5 details the research questions the present study addresses.

#### 2.3.1 Modulating the verb sign

Across sign languages, it has been observed that grammatical aspectual distinctions can be encoded by modulating or inflecting4While modulating the verb sign to express aspectual distinctions has often been analyzed as an instantiation of inflection (e.g., Klima and Bellugi 1979; Rathmann 2005), Bergman and Dahl (1994) offer a different analysis for Swedish Sign Language (SSL). They argue that the reduplication system of SSL differs from inflectional processes in spoken languages (they mention obligatoriness and lexical generality as essential properties of inflection, lacking from the reduplication system in SSL), but rather shares many properties with ideophonic components in spoken languages. Here, I analyze verbal modulation as inflection, and refer to Bergman and Dahl (1994) for the alternative analysis. the verb sign, or, more specifically, modulating the verb’s movement. Klima and Bellugi (1979) (building on work by Fischer [1973]) were the first to provide an extensive overview of 15 different aspect types in American Sign Language (ASL), which are distinguished by modulating the verb. Examples of such modulations include reduplication, changing the rate of signing, and/or adding pauses in between reduplication cycles – these modulations are argued to result from morphological processes applying to the verb sign.

The list of 15 ASL aspect types was later brought back to six aspect types by Rathmann (2005). This comprehensive study also provides evidence for the morphemic status of the distinguished aspect types in ASL. Five of these aspect types are encoded by modulating the verb sign, among which the continuative, iterative, and habitual aspect, which are defined in a similar way as introduced above for spoken languages. While such inflections are typically subsumed under grammatical aspect (see, e.g., Quer et al. 2017), Rathmann (2005) argues that in ASL, four of them (continuative, iterative, habitual, and hold) belong to the situation-type component rather than the perfective or imperfective viewpoint.5Rathmann (2005: 174) gives two reasons for this: (i) these aspectual morphemes can co-occur with perfective finish, and (ii) they are concerned with duration, telicity, and dynamism. In the present study, however, I follow previous work (Comrie [1976] on spoken languages and Quer et al. [2017] on sign languages, among many others) in assuming that inflections expressing the habitual and continuative are generally instances of imperfective aspect, and that inflections expressing the iterative are generally instances of perfective aspect, and as such that they are grammatical aspect (but see Section 5.2 for further discussion).

Continuative aspect – which Rathmann (2005: 36) defines as “the temporal interval over which the eventuality unfolds is longer than usual and uninterrupted”, as in (4a) – is expressed in ASL by extending the movement of the verb root for a longer time than in the citation form. Iterative aspect – which he defines as “multiple instances of the eventuality unfold in their own intervals” (p. 39), as in (4b) – is expressed by reduplication of the verb’s movement. Finally, habitual aspect is defined as “there is a property that is characterized by a regular repetition of the eventualities and that holds over an interval of time” (p. 42), as in (4c). This is also expressed by reduplication of movement, but in shorter and quicker cycles than the iterative morpheme. The glossing conventions for the sign language examples are given in Appendix A.

(4)

a.

**Table:** 

today, mary cook, john cook+continuative
‘Today, Mary cooked, but John cooked even longer.’
(ASL; Rathmann 2005: 35)

b.

**Table:** 

john cook+iterative
‘John cooked repeatedly.’
(ASL; Rathmann 2005: 38)

c.

**Table:** 

john go+habitual church
‘John goes to church (regularly).’
‘John usually goes to church.’
(ASL; Rathmann 2005: 41)

Apart from ASL, modulating the verb sign to encode aspectual distinctions has been observed for several other sign languages. For example, in British Sign Language (BSL), the continuative form of verbs without a path movement is expressed by an extended hold (Sutton-Spence and Woll 1999), and repetition of movement was found to encode iterative aspect in Indo-Pakistani Sign Language (IPSL) (Zeshan 2000) and habitual aspect in Spanish Sign Language (LSE) (Cabeza Pereiro and Fernández Soneira 2004), among others. Gray (2013), in his extensive corpus study on aspect in Australian Sign Language (Auslan), finds different types of reduplication (in a “fast, unmarked or slow manner” [p. 142]). However, unlike what has been described in the other studies mentioned here, Gray (2013: 159) notes that these different reduplication types “do not appear to form a clearly defined paradigm of discrete, categorical options”. Rather, according to Gray, the specific meaning is determined by the context, and, for instance, whether the reduplication co-occurs with constructed action. He notes that the number of repetitions in reduplication varies, and a larger number may reflect a more frequent repetition of the event.6
Gray (2013) thus observes no fixed and predictable forms, and no completely consistent semantics in reduplication expressing aspectual meanings in Auslan. Reduplication is also not obligatory to describe repeated or continuing events. Given these – among other – observations, Gray (2013) argues against the existence of a set of aspectual morphemes with fixed forms, and against an analysis of Auslan aspect marking as an inflectional morphological system. Instead, Gray analyzes aspectual modification in Auslan as gestural modification of verbs (for details, I refer to Gray [2013]).


The encoding of aspect in sign languages does not always involve marking on the predicate: free-standing markers are also attested, to which we turn now.

#### 2.3.2 Free-standing aspectual markers

Rathmann (2005) notes that one of the six aspectual distinctions in ASL is encoded by a free-standing aspectual marker: a sign glossed as finish, which expresses that “the eventuality is bounded” (p. 48); i.e., perfective viewpoint. Cross-linguistically, such free-standing markers often indicate that an event is finished, i.e., perfective or completive aspect, as is true, for example, for already in Israeli Sign Language (ISL) in (5) (Meir 1999).

(5)

**Table:** 

index_1_ **already** write letter sister poss_1_
‘I have written a letter to my sister.’
(ISL; adapted from Meir 1999: 51)

Such markers have also been identified, for instance, for German Sign Language (Rathmann 2005), SSL (Bergman and Dahl 1994), ASL (Fischer and Gough 1999; Janzen 1995), Turkish Sign Language (Karabüklü and Wilbur 2021; Zeshan 2003), and IPSL (Zeshan 2003), and Johnston et al. (2015) describe the ongoing grammaticalization of different signs glossed as finish in Auslan.

Manual perfective markers are sometimes accompanied by non-manual elements, that is, silent articulations by the mouth. For instance, for Kata Kolok (Bali), De Vos (2012a, 2012b) describes that the manual sign finish is often accompanied by a non-manual element ‘pah’. She observes that the non-manual element can also attach to a lexical predicate to express the perfective, and in those cases, it occurs without finish. Similarly, for the urban sign language varieties of Solo and Makassar (Indonesia), Palfreyman (2013) finds that completive aspect can be encoded by at least four particles, which may co-occur with completive-marking mouthings. He observes that these mouthings may co-occur with the (manual) particle, but they, too, can also occur as the only completive marker in the sentence (see also Palfreyman 2015). For Turkish Sign Language, Karabüklü and Wilbur (2021) find that, apart from the manual marker bİt (which is similar to ASL finish), the non-manual marker ‘bn’ is a separate perfective morpheme.

Free-standing manual signs have been described for other types of aspect as well, albeit less frequently. For instance, in SSL, a habitual marker (which is not obligatory) has been identified; use of this marker, which is glossed as usually, is illustrated in (6). Note that here, usually co-occurs with reduplication of the verb.

(6)

**Table:** 

**usually** sit write++ letter
‘He usually [sits down and] writes letters.’^7^
(SSL; Bergman and Dahl 1994: 402)

7 This is a free translation, as Bergman and Dahl (1994) do not offer a translation for this example.

Free-standing aspect markers have also been identified for NGT (Hoiting and Slobin 2001; van Boven 2018; van Boven and Oomen 2021), as I will discuss in the following section.

### 2.4 Previous studies on aspect in NGT

The first to describe the encoding of aspectual distinctions in NGT were Hoiting and Slobin (2001), focusing on habitual and continuative aspect in this language. According to them, continuative aspect describes an action that is ongoing (e.g., “He’s going on working [at the moment]” [p. 128]), and is marked on the verb sign by “three repetitions of an elliptical modulation accompanied by pursed lips and a slight blowing gesture” (p. 127). Habitual aspect, which they define as describing an ongoing action that occurs habitually (e.g., “He always works on and on” [p. 128]), is marked on the verb sign by “a slower elliptical modulation accompanied by gaze aversion, lax lips with protruding tongue, and slowly circling head movement” (p. 127). Note that both definitions are somewhat unconventional: in the definition of continuative aspect, there is no reference point (e.g., Klein’s [1994] “topic time”; see the definition given in Section 2.2), and in the definition of habitual aspect, there is a notion of continuity, implied by the word ‘ongoing’ – traditional definitions of habituality (and the one we adopt in the present study), however, do not include such a notion (see, again, Section 2.2). Hoiting and Slobin do not provide examples of the elliptical modulation. Yet, they do identify phonological constraints.

For the reader to appreciate these constraints, it is necessary to introduce the sublexical (i.e., phonological) building blocks of signs, which have been argued to function like segments in spoken languages (sometimes called “parameters”). Simplifying somewhat, the building blocks that have been identified are the handshape (hand configuration), place of articulation, and movement of the sign (Sandler 1989; Stokoe 1960; for an overview, see Fenlon et al. [2017]). The latter comes in two types: path movement and hand-internal movement. If a sign has a path movement, the hands move from one location to another. Internal movement involves a change in handshape or in orientation. Another kind of internal movement is so-called secondary or trilled movement, which involves fast repetitions of orientation or handshape changes, or wiggling of the fingers. Internal movement and path movement may combine, or occur on their own (Brentari 1990; Sandler and Lillo-Martin 2006; Wilbur 1987). Evidence for the phonological significance of these building blocks comes from minimal pairs; for instance, two signs may differ in terms of their movement alone. Furthermore, just like segments in spoken languages, these building blocks can be described in terms of distinctive features, which are organized in feature hierarchies. Various phonological models have been put forward (e.g., Brentari 1998; Sandler 1989), to which I will return in Section 5.1.

**Figure 1:**
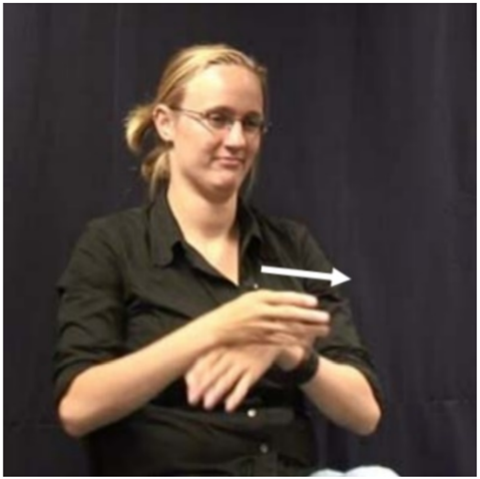
cont in NGT (van Boven 2018: 7), video still taken from the Corpus NGT (Crasborn et al. 2008; Crasborn and Zwitserlood 2008).

According to Hoiting and Slobin (2001), aspectual marking in NGT is phonologically constrained, the relevant building blocks being movement and place of articulation: verbs with internal movement and/or body contact cannot undergo the elliptical modulation. These verbs thus remain uninflected, but are followed by an aspectual particle which they gloss by means of the Dutch word door (lit. ‘through’), which takes on the inflection instead. This particle is borrowed from spoken Dutch, where it can be used with a similar meaning with some verbs (e.g., *Hij loopt door* ‘He continues to walk’). The sign is illustrated in Figure 1. In the following, I will gloss this sign as cont (since later studies have shown that this particle expresses continuity, but not habituality, as we will see below). An example where a verb with body contact (try, which contacts the nose) is combined with cont, which then takes on the inflection, is given in (7).

(7)

**Table:** 

index_3a_ try **cont++**
‘He tried continuously.’
‘He tried and tried and tried.’
(NGT; adapted from Hoiting and Slobin 2001: 129)

A few recent studies have called into question the analysis of continuative and habitual marking offered by Hoiting and Slobin. First, Oomen (2016) conducted an elicitation task with one native signer. This task followed the general format of Dahl’s (1985) questionnaire, originally developed to elicit tense, mood, and aspect (TMA) in spoken languages, but which was later also used to investigate SSL (Bergman and Dahl 1994). This questionnaire consists of approximately 200 sentences preceded by a question or context sentence that is supposed to trigger specific TMA marking (if available in the language); the verbs in the target sentences are given in their infinitival forms as in (8). Sentences are presented in English and informants are asked to translate the sentences to a target language (Dahl 1985).

(8)

**Table:** 

Question:	What your brother usually DO after breakfast?
Answer to be translated:	He WRITE letters
(Dahl 1985: 199)	

Oomen (2016) used this questionnaire in slightly adapted form: she brought the number of sentences down to 66, translated the sentences into Dutch, and adapted the items to the purpose of her study, such that they would (i) trigger continuative and habitual aspect and (ii) target verbs that were selected based on their phonological specifications.

Oomen’s results suggest that both aspect types are expressed in NGT by reduplication combined with non-manual markers. Specifically, continuative aspect was “consistently marked by means of a relatively slow reduplication of the verb’s movement and a synchronous back-and-forth movement of the head or body” (Oomen 2016: 43), while habitual aspect involved reduplication and synchronous left-to-right head and body movements. Habitual marking only occurred in past contexts. Notably, cont did not appear in the data at all: verbs with internal movement or body contact were inflected for continuative and habitual aspect in the same manner as those without these constraining features. Oomen offers regional variation as a potential explanation for the differences between these two studies: she reported on an informant from Amsterdam, while Hoiting and Slobin included informants from Groningen. It is well-known that there are lexical differences between the NGT dialects from these regions (Schermer 2004), but Oomen is the first to suggest a grammatical (i.e., inflectional) difference. It is also important to note that Oomen defined habituality in a more traditional way than Hoiting and Slobin, that is, excluding the notion of continuity.

In a subsequent study, van Boven (2018) presents a descriptive analysis of cont based on naturalistic corpus data, searching for this sign (i.e., the gloss door) in the Corpus NGT (Crasborn et al. 2008; Crasborn and Zwitserlood 2008). The results again challenge Hoiting and Slobin’s description, as well as Oomen’s observations: cont does occur in the data, but it co-occurs with a wide variety of verbs, not only with those involving body contact or internal movement. It also appears in various syntactic positions, sometimes preceding rather than following the verb. cont was mostly used to express continuative aspect.

Finally, habitual aspect in NGT was further investigated by van Boven and Oomen (2021). They, too, investigated naturalistic corpus data from the Corpus NGT. They searched for any sentence that refers to an event that is regularly repeated over an extended period of time. They conducted specific searches in the Dutch translations available in the corpus, searching for particles and adverbials that normally occur in Dutch habitual sentences (e.g., *regelmatig* ‘regularly’, *per week* ‘a week’, and *elke dag* ‘every day’). In total, they included 106 sentences in their data set, which, according to them, have habitual meaning. They then analyzed how this habitual meaning is encoded in the sentence. Since the present study builds on their methodology, and actually includes these data, more details about the search procedure are given in Section 3.1.

While they could not identify clear non-manual markers, van Boven and Oomen found that habitual meaning can be realized by reduplicating the verb, as well as by several adverbials. These two types of manual markers sometimes occur together (26.4 % of the 106 analyzed sentences), but also on their own (40.6 % for adverbs; 19.8 % for reduplication). Moreover, manual marking is not obligatory (13.2 % of the sentences does not involve any manual marking). (9a) shows a sentence with both reduplication and the adverbial always, while (9b) illustrates a sentence without any manual marking.

(9)

a.

**Table:** 

index_3a_ **always one time month angry++**
‘He always used to be angry once a month.’
(NGT; van Boven and Oomen 2021: 170)

b.

**Table:** 

say past index_1_ certain
‘I used to say “I’m certain [it will forever stay that way]”.’
(NGT; van Boven and Oomen 2021: 169)

As for cont, van Boven and Oomen (2021) argue that it does not express habitual meaning: it occurs in only two out of 106 sentences, and in both cases, it triggers an additional continuative reading. Therefore, they conclude that cont actually encodes continuative rather than habitual aspect, which aligns with the fact (i) that Hoiting and Slobin (2001) maintained a definition of habituality that includes the notion of continuity, and (ii) that van Boven (2018) found this sign to mostly express continuity.

In the corpus data, habitual marking occurs with verbs with a variety of phonological properties, as well as both in past and non-past contexts. Van Boven and Oomen offer a methodological explanation for the differences between their study and the previous studies on NGT: corpus data display more variation than elicited data.

### 2.5 The present study

Strategies for encoding habitual and continuative aspect in NGT have been investigated previously. From these studies, it seems clear that reduplication plays a role. Yet, many questions remain unanswered, since there is considerable variation in the results. Moreover, none of the previous studies takes into account iterative aspect, while in other sign languages, iterative aspect has often been found to be encoded by reduplication as well. The present study therefore aims at answering the following questions:

(I) Can reduplication of the predicate encode habitual, continuative, and iterative aspect in NGT?
(II) Can different types of reduplication be distinguished for different types of aspect in NGT?
(III) Which restrictions on aspectual reduplication can be identified in NGT?
  - Are there phonological restrictions (internal movement/body contact)?
  - Is there a relation between cont and reduplication for continuative aspect?
  - Is there a relation between tense and reduplication for habitual aspect?

Previous studies on aspect in NGT included only one (Oomen 2016) or a few informants (Hoiting and Slobin 2001) from one sign region and focused only on elicited data that sometimes involved translations – and thus potential influence – from spoken Dutch (Oomen 2016). Some studies included naturalistic corpus data (van Boven 2018; van Boven and Oomen 2021), but none of the previous studies on NGT systematically analyzed all three aspect types that are potentially expressed by reduplication. Therefore, the current study is the first to comprehensively analyze and compare all three aspect types, combining naturalistic corpus data with data elicitation, in order to provide a comprehensive description of aspectual reduplication in the language.

## 3 Methods

The method of this study is twofold: first, building on van Boven and Oomen (2021), data were collected from the Corpus NGT (Section 3.1), and second, data were elicited from deaf informants (Section 3.2). Both data sets were annotated and analyzed in a similar manner (Section 3.3).

### 3.1 Corpus search

The starting point of this study was a search in the Corpus NGT (Crasborn et al. 2008; Crasborn and Zwitserlood 2008), which contains recordings of 92 deaf NGT signers (age 17–84 years). The signers in the corpus performed several tasks, such as discussing certain topics and retelling video clips, resulting in semi-spontaneous monologues and dialogues.8For more information on the Corpus NGT, such as the elicitation materials, metadata, and all public corpus files, see https://archive.mpi.nl/tla/islandora/object/tla:1839_00_0000_0000_0004_DF8E_6?asOfDateTime=2018-03-02T11:00:00.000Z. The corpus contains over 70 h of video data, and part of these data have been transcribed by fluent signers, using the annotation tool ELAN (Crasborn and Sloetjes 2008). The available transcriptions include annotations on gloss tiers, where signs are glossed on separate tiers for the dominant and non-dominant hand, and a translation tier, which includes Dutch translations (Crasborn et al. 2008).

The present study includes the corpus data collected for habitual aspect by van Boven and Oomen (2021), as reported above. In order to complement these data with constructions involving iterative and continuative aspect, I adopted the same strategy, i.e., I included sentences in my data set based on their meaning: the Corpus NGT is not annotated for aspectual distinctions or reduplication, and for this reason, searches had to be conducted on the translation tier, searching for particles and phrases that often occur in Dutch continuative and iterative sentences. Additionally, for continuative aspect, one specific gloss was searched for (hele ‘whole’), as this sign was expected to often occur in continuative sentences (e.g., in the context of the phrase hele dag ‘the whole day’). Table 1 provides an overview of the specific search terms, and specifies how many search hits there were in total per aspect type,9Please note that these are not all unique hits, as some sentences in the corpus showed up for multiple search terms (e.g., a sentence that contains both *voortdurend* ‘continuously’ and *de hele dag* ‘the whole day’ would surface twice as a search hit). and how many sentences were included in the end for each aspect type.

**Table 1:** Search terms used to find different aspectual distinctions in the Corpus NGT. The searches were conducted on the translation tier, with one exception (hele ‘whole’ on the gloss tier for the continuative).

Aspect type	Search terms	N (total hits)	N (hits included)
Habitual (see van Boven and Oomen 2021)	*regelmatig* (‘regularly’) *iedere/elke dag/week/jaar/maand* (‘each day/week/year/month’) *per dag/week/jaar/maand* (‘per day/week/year/month’) *altijd* (‘always’) *vaak* (‘often’) *elk(e)*, *ieder(e)* (‘each’)	218	106
Continuative	*aan het* (locative expression to indicate continuative meaning, lit. ‘at the’) *voortdurend* (‘continuously’) *zit te/zitten te/zat te/zaten te* (light verb construction to indicate continuative meaning, lit. ‘sit/sat to’) *hele dag/heel de dag* (‘whole day’) *continu* (‘continuously’) *door* (postposition or verb particle to express continuity, lit. ‘through’) *constant* (‘constantly’) *hele* *(gloss)* (whole)	520	106
Iterative	*opnieuw* (‘again’) *herhaaldelijk* (‘repetitively’) *steeds* (‘always’) *elke/iedere keer* (‘each time’) *keer* (‘time’) *vaak* (‘often’)	269	28

Each sentence that potentially involved the relevant aspect type was included. The inclusion criteria for the habituals are explained in van Boven and Oomen (2021: 165–166), but are briefly repeated here. Adopting definitions of habituals by Comrie (1976) and Rathmann (2005), van Boven and Oomen searched for any sentence that references an event that is regularly repeated over an extended period of time (see also the definitions in Sections 2.2 and 2.3). As Table 1 shows, 106 sentences met this criterion, and representative examples are given in (10) (van Boven and Oomen 2021: 166). For all of the examples extracted from the corpus, the corpus file number (CNGTxxxx), signer number (Sxxx), and begin time of the example (m:s.ms) are provided.

(10)

**Table:** 

*Examples included in the habitual data set* (van Boven and Oomen [2021: 166]; part.aff = affirmative particle)

a.

**Table:** 

index_1_ go++ part.aff palms.up	
‘I go there regularly.’	
[CNGT0064; S006; 01:56.90]

b.

**Table:** 

one time day go beach […]	
‘Every day we went to the beach […].’	
[CNGT0049; S006; 04:06.20]

Table 1 also shows that 112 sentences showed up in the searches for habituals, but did not meet the inclusion criteria – i.e., they did not involve a systematically recurring event, and therefore were excluded. Two representative examples are given in (11) (van Boven and Oomen 2021: 166). Both showed up in the searches because the Dutch translations contain *altijd* ‘always’.

(11)

**Table:** 

*Examples excluded from the habitual data set* (van Boven and Oomen 2021: 166)

a.

**Table:** 

always index_3_	
‘Was he always like that?’	
[CNGT0370; S019; 00:57.20]

b.

**Table:** 

index_3_ book read always important index_3_	
‘And that – reading books – is always important.’	
[CNGT0429; S021; 02:59.50]

For continuative aspect, I adopted the definition given in Section 2.2, i.e., the sentence was included when “an event is progressing dynamically over a time frame opened up by an utterance” (Mair 2012: 803), and also when a situation is in progress. In total, 106 sentences met these criteria, as Table 1 shows. Two representative examples are given in (12).

(12)

**Table:** 

*Examples included in the continuative data set*

a.

**Table:** 

continue index_1_ write
‘I continued to write.’
[CNGT0121; S007; 00:24.92]

b.

**Table:** 

index_3a_ talk cont++
‘She (the cashier) just continued talking.’
[CNGT0134; S008; 01:20.64]

As is evident from Table 1, 414 sentences showed up in the searches for continuative aspect but did not meet the criteria, and were thus excluded. Representative examples are given in (13) – these do not involve ongoing, progressing events or situations. These sentences showed up in the search hits because their Dutch translations contained *(koppelen) aan het* ‘connect to’ (13a) and *(ingehaald) door* ‘caught up with by’ (13b).

(13)

**Table:** 

*Examples excluded from the continuative data set*

a.

**Table:** 

same start write read connect speech-therapy have-to
‘You can connect speech therapy to reading and writing.’
[CNGT0255; S013; 05:50.88]

b.

**Table:** 

stop police go-away quick
‘Then we were quickly caught up with by the police.’
[CNGT0050; S006; 00:35.28]

Iterative aspect had not previously been investigated for NGT. Therefore, I assumed the definition proposed by Rathmann (2005: 37), which is also given in Section 2.2.1 above: “The iterative morpheme contributes the meaning that multiple instances of the eventuality unfold in their own interval. A break is possible between each interval”. It was thus important that an event occurred multiple times, each instance with its own starting point (similar definitions are given in, e.g., Comrie [1976] and Bybee [1985]). In total, 28 sentences met these criteria (see Table 1), and representative examples are given in (14).

(14)

**Table:** 

*Examples of sentences included in the iterative data set*

a.

**Table:** 

some people try set-up+
‘Some people tried to set something up over and over again.’
[CNGT0259; S014; 04:16.88]

b.

**Table:** 

sleep++ drive-car
‘My eyes kept closing [I kept falling asleep] while driving the car.’
[CNGT0050; S006; 00:32.56]

However, the vast majority of the sentences that showed up in the searches for iterative aspect, 241 in total (see Table 1), did not involve multiple occurrences of an event and were therefore excluded from the data set. Two representative examples are given in (15); both found their way into the search hits because the Dutch translations contain *opnieuw* ‘again’.

(15)

**Table:** 

*Examples of sentences excluded from the iterative data set*
headshake

a.

**Table:** 

pleasant new learn index_1_ agree
‘It wasn’t pleasant to learn that [sign] again // to learn a new sign.’
[CNGT0069; S006; 03:33.8]

b.

**Table:** 

[…] again start-up […]
‘[…] and it (the computer) restarts […]’
[CNGT1721; S071; 00:16.6]

### 3.2 Data elicitation

In order to complement the corpus data, an elicitation task was designed, aimed at eliciting NGT sentences that contain continuative, habitual, and iterative aspectual marking.

#### 3.2.1 Participants

Six deaf NGT signers participated in this study (none of which are included in the Corpus NGT). All signers grew up with NGT. Their mean age is 41 years (range 27–67), two are male and four are female, and they come from various sign regions in the Netherlands (one from Zoetermeer, two from Groningen, two from Amsterdam, one from mixed regions). While three of them have deaf family members, the other three grew up with only hearing family. Data from one additional participant had to be excluded from the analysis, as this participant did not carry out the test as it was intended.

#### 3.2.2 Stimuli

The elicitation task aimed at eliciting aspect marking on six different NGT verbs with different phonological features, as shown in Table 2.10In practice, participants sometimes used different variants of these verbs with different phonological features – for example, for melt, a variant without internal movement but with body contact was sometimes used, as will become clear below. For each verb, participants were presented with six items (i.e., there were 36 stimuli in total): two for each aspect type, once in a non-past context and once in a past context (note that NGT does not mark tense on the verb; rather, the tense information was provided by time adverbials such as yesterday).

**Table 2:** Verbs included in the elicitation task.

Verb type		
Body-anchored	Internal movement	No potentially constraining features
sleep	melt	clean
hug	talk	swim

**Figure 2:**
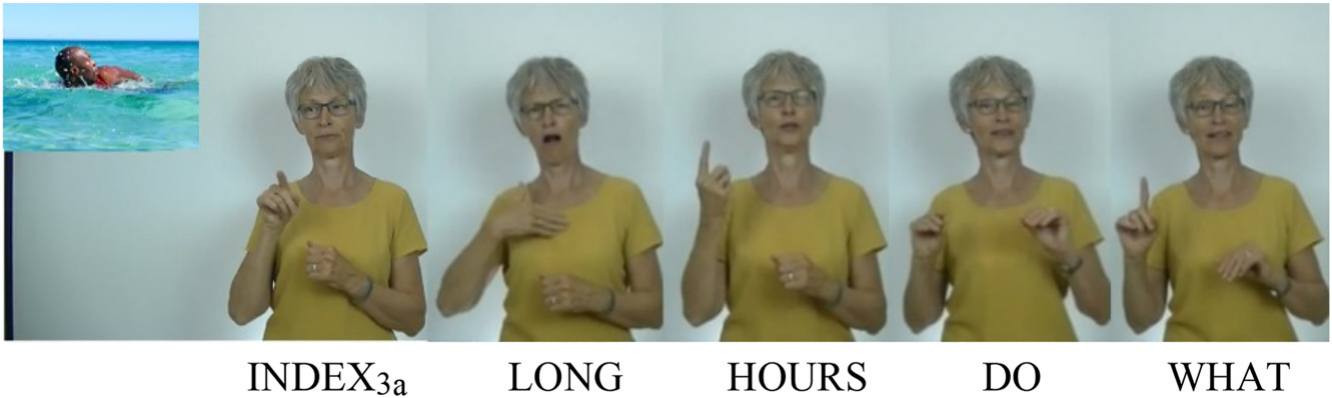
Example of an elicitation item: “[This man lives close to the beach.] What has he been doing for hours?”. Targeted answer: “The man has been swimming for hours.” (Stimulus picture from Dreamstime.com, Image ID: 75474659, Copyright Mimagephotography, https://www.dreamstime.com/mimagephotography_info).

Each elicitation item consisted of two parts: (i) a picture denoting the verb, for instance, a man who is swimming, and (ii) a question in NGT about that picture, preceded by a brief context, for instance, “This man lives close to the beach. What has he been doing the past few hours?”; see Figure 2.

Participants were asked to answer the question based on the picture they saw. Importantly, the question was phrased such that it would elicit an answer that contains marking for one of the aspectual distinctions under investigation. That is, the preceding context and the question specify how often a certain event occurs and/or how long the event lasts. Neither the context nor the question contained the target verb (in the example “to swim”), in order to avoid influencing the answers as much as possible. Participants were instructed to answer in complete sentences (in our example, an expected answer would be translated as ‘The man has been swimming for hours’).

Additionally, the base form of each verb was elicited (six items in total), in order to be able to compare the verbs in the sentences potentially marked for aspect to their base form articulated by the same participant. This was done by simply asking what the person on the picture is doing. Therefore, in total, the participants were presented with 42 elicitation questions and pictures. An English translation of the elicitation task, including instructions, is openly available (van Boven 2023). All items and instructions were presented by a deaf NGT signer, who was also consulted when designing the elicitation items.

Some of the contexts presented to the participants were based on the TMA-questionnaire developed by Dahl (1985) and adapted for NGT by Oomen (2016). That is, I based the items for continuative and habitual aspect on the contexts in this questionnaire for those verbs that can be depicted on a picture (i.e., sleep, hug, talk, and clean) – although where necessary, some changes were made – and complemented these with two new verbs (melt, swim), as well as new contexts for iterative aspect.

The stimuli were piloted with one deaf NGT signer, after which six stimuli were adapted since they did not elicit the targeted aspect types. The adaptations were done in consultation with a deaf signer.

#### 3.2.3 Procedure

After the pilot, six other signers participated in the task, which was designed in Qualtrics.11Available at https://www.qualtrics.com. Before the actual task started, they provided their informed consent, allowing for the use of the gathered data and video stills. They were then presented with a few background questions (age, sex, sign region, hearing status of family members, and the languages they know). Subsequently, a deaf signer provided instructions for the task in NGT, which, importantly, mentioned that participants should use entire sentences to answer the questions about the pictures (that is, not just the verbs). They were then shown two example items and answers featuring verbs that were not included in the actual task. The example items did not involve aspectual meaning. Then the actual test started.

Four participants (of which one had to be excluded) came to the recording studio to take part in the study, while the other three participants preferred to take part via a videocall. In all cases, a hearing researcher (who signs NGT as a second language) was present, either in person or via the videocall platform.

The 42 stimuli were presented in a semi-randomized order that was different for each participant. Within the randomization, it was ensured that items targeting the same verbs, the same aspect types, and the same tenses did not follow each other. Stimuli were presented one by one, and after watching a stimulus, participants signed their answer to the camera (either on their laptop or the camera in the recording studio). They could then move on to the next stimulus, sign their answer, and so on. There was no time pressure; participants could decide themselves when they wanted to move on to the next elicitation item. The hearing researcher only answered any practical questions a participant might have and did not interfere during the task.

### 3.3 Data annotation and analysis

The data annotation was done for both data sets in ELAN (Crasborn and Sloetjes 2008) – see Section 3.3.1. The statistical analyses were conducted in R (R Core Team 2008) and are introduced in Section 3.3.2.

#### 3.3.1 Data annotation

**Figure 3:**
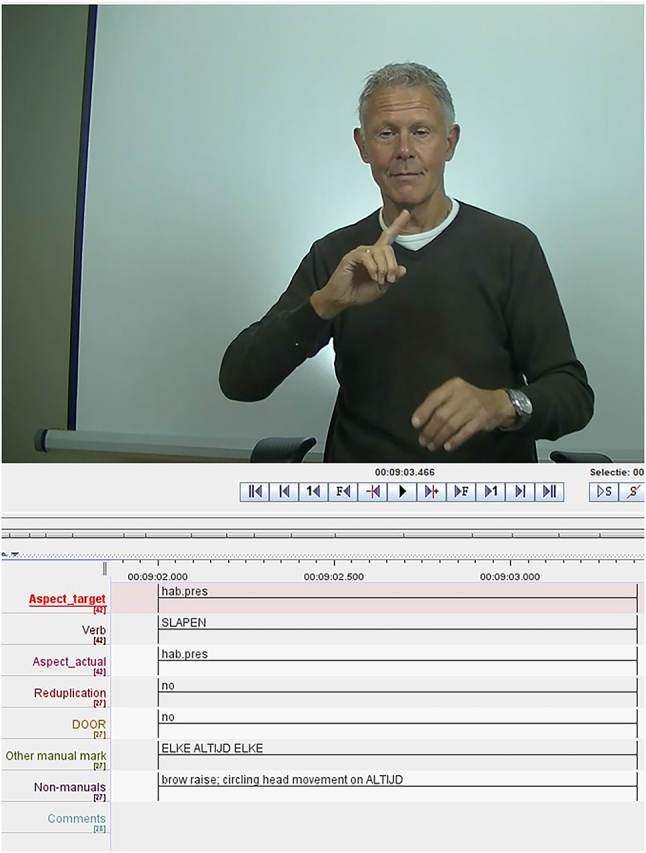
Example of the annotations in ELAN.

The annotation tool ELAN (Crasborn and Sloetjes 2008) was used to annotate both data sets. To illustrate, a screenshot of the annotations is shown in Figure 3. For both data sets, I annotated (i) the aspect type of the sentence, (ii) whether the predicate was reduplicated, (iii) whether the sentence contained an adverb marking the aspect type, (iv) any non-manual markers potentially expressing aspect, and (v) any additional comments or relevant information about the phonological features of the predicate. For the elicited data, I additionally annotated (vi) any difference between the targeted aspect type and the aspect type actually produced by the participant, (vii) whether the sentence is past or non-past, and (viii) whether the participant added cont to the sentence. All data annotations for both data sets are openly available (van Boven 2023).

For the habituals in the corpus data, I adopted the relevant annotations already made by van Boven and Oomen (2021) (i.e., on reduplication, adverbs, non-manual markers, past tense, and comments).12I adapted two of the annotations originally made by van Boven and Oomen (2021). These involve two habitual sentences with the predicate get.used.to (Dutch gloss wennen, [CNGT1627; 00:50.8] and [CNGT1926; 01:22.1]). Originally, van Boven and Oomen annotated that this predicate was reduplicated, but upon further analysis I decided to annotate that it was not. In its citation form, get.used.to has an inherently repeated movement – in the NGT dictionary (https://www.gebarencentrum.nl/Gebarenwoordenboek) it has four movement cycles. Both corpus instances have only two movement cycles, even less than the citation form, and therefore they are now subsumed under inherent repetition, and no longer annotated as reduplicated. I added annotations of the relevant phonological features.

As for the elicited data, I did not only annotate productions including the targeted verbs, but also sentences in which participants produced a verb that was not targeted, but which still involved one of the aspects. Sometimes, participants produced the targeted verb meaning, but used a different variant of that verb than was expected (e.g., the target form of melt involves internal movement, but some participants produced a variant without internal movement, but with body contact; see also Footnote 10) – these were also included.

#### 3.3.2 Statistical analyses

A statistical analysis was conducted to investigate under what circumstances aspectual reduplication occurs (i.e., research questions (I) and (III)), specifically whether (i) the aspect types differ with respect to each other in terms of reduplication (in both data sets) (ii) the data sets (i.e., the corpus and the elicited data), differ from each other in terms of reduplication, and (iii) there is an interaction between aspect type and data set. The data were trimmed: I excluded elicited verbs that could not be analyzed for reduplication (see Section 4.1).

I fitted a generalized linear mixed effects models using the lme4 package (Bates et al. 2015). I built a model with reduplication as the dependent variable, a binomial factor with two levels: ‘1’ (when the predicate is reduplicated) or ‘0’ (when the predicate is zero-marked). Aspect type and data set were included as fixed effects, and an interaction between them was included. With the aim to fit a maximal model justified by the design (Barr et al. 2013), a random intercept for subject was also included as well as a by-subject random slope for aspect type.

I used orthogonal sum-to-zero contrast coding for the aspect type variable. I set the following comparisons: (a) continuative and habitual against iterative (contrast coded as −1/3, −1/3, +2/3, respectively); (b) continuative against habitual (contrast coded as −0.5, +0.5, respectively, with iterative coded as 0). I also used orthogonal sum-to-zero contrast coding for the data set variable. Corpus data was coded as +0.5 and elicited data as −0.5.

An important qualification, however, is that the analysis of binomial data, such as the data analyzed here, is known to require large samples. For instance, Moineddin et al. (2007), in their simulation study, report that multilevel logistic regression models require at least 50 groups, with a group size of 50, in order to produce valid estimates, and that in each group the expected number of outcomes should be more than one. When using logistic regression on smaller data sets, “researchers can expect to encounter convergence problems, large biases in their model estimates and inadequate statistical inference procedures. Our findings suggest that when choosing a sample size, researchers should base their decision on the level of bias that they consider acceptable for that particular study” (Moineddin et al. 2007: Discussion and conclusion, para. 11). Given that the data set analyzed here involves 363 observations of 55 participants (corpus and elicited data together), the estimates reported here should be taken with caution. See Section 5.3 for more discussion.

Further, for the elicited data, it was analyzed statistically whether there is a relationship between reduplication and the predicates’ potentially constraining phonological features, as well as between reduplication and past tense. For this, the data were trimmed, too, excluding again elicited verbs that could not be analyzed for reduplication, as well as the corpus data. Given the fact that this involves an even smaller sample size (123 observations of six participants), no logistic regression model was conducted. Instead, I conducted Pearson Chi-Squared Tests in R (R Core Team 2008). However, we should bear in mind that a chi-squared test assumes that the observations in the data set are independent of each other (see, e.g., Agresti [2007] for more information about chi-squared analysis), which is not the case in the present data sets. The results could, in fact, be due to several dependencies in the data (for instance, the fact that there are multiple measurements per participant), and this should be kept in mind when interpreting the results. See Footnote 13 and Section 5.3 for more discussion.

Finally, a Pearson Chi-Squared Test was also conducted in R (R Core Team 2008) to investigate whether there is a relationship between the occurring aspect type and the data set. Again, the same caveat as mentioned above should be kept in mind.

The statistical analyses (a .Rmd-file and an html version) are openly available (van Boven 2023).

## 4 Results

In total, 240 sentences in the corpus were analyzed as involving one of the three targeted aspect types. In addition, 172 sentences were elicited; see Table 3 for an overview.

**Table 3:** Habitual, continuative, and iterative sentences in the Corpus NGT and the elicited data.

Aspect type	Corpus (N)	Elicited (N)
Habitual	106^a^	63
Continuative	106	47
Iterative	28	62
Total	240	172

^a^See van Boven and Oomen (2021).

Table 3 makes clear that all aspect types occur in both data sets, but that iterative aspect is clearly underrepresented in the corpus data. The Pearson Chi-Squared Test I conducted in R (R Core Team 2008), in order to investigate the relation between aspect type and data type (i.e., Table 3), shows a significant relation between the two variables (*X* ^2^ (2, *N* = 412) = 36.3, *p* < 0.001).13However, we should bear in mind that a chi-squared test assumes that the observations in the data set are independent of each other (see, e.g., Agresti [2007] for more information about chi-squared analysis), which is not the case in the present data sets. The result could, in fact, be due to several dependencies in the data, for example, because certain participants differ from others (there are multiple measurements per participant). However, it is likely that the significant result is due to the fact that in the elicited data, I targeted an equal amount of sentences for each aspect type, likely resulting in an unusually high number of iteratives as compared to the naturalistic language use in the corpus data. I refer to Section 5.3 for further discussion of this difference in frequency. An overview of all statistical data is provided in Appendix B, and see van Boven (2023) for the complete analysis in R.

### 4.1 Reduplication of the predicate

Table 4 shows how often reduplication is used to express an aspectual distinction in the corpus data. Clearly, reduplication of the predicate occurs, but not always: for habitual and continuative sentences, about half of the predicates is reduplicated in the corpus data, while for iteratives in the same data set about 30 % of the sentences does not involve a reduplicated predicate.

**Table 4:** Reduplication encoding aspectual meaning in the corpus data.

Aspect type	N (total)	Predicate reduplicated
Habitual (van Boven and Oomen 2021)^a^	106	47 (44.3 %)
Continuative	106	56 (51.9 %)
Iterative	28	20 (71.5 %)
Total	240	123 (51.3 %)

^a^Recall from Footnote 12 that I re-analyzed two instances that van Boven and Oomen (2021) originally analyzed as predicate reduplication. The number in Table 4 for reduplication in habituals in the corpus is therefore slightly lower than in their analysis (47 rather than 49 instances).

For the elicited data, some sentences could not be analyzed for reduplication, since for some predicates, no corresponding base form produced by the same participant could be elicited – be it because the elicitation of the base form did not succeed, or because the participants inflected a verb for aspect that was different from the targeted verb. For this reason, 14 continuative, 25 habitual, and 10 iterative sentences could not be analyzed for reduplication. Table 5 shows the results for reduplication of the predicate in the remaining elicited sentences – for habitual and continuative sentences in the data, about 20 % involves predicate reduplication, while this percentage is, again, about 70 % for iterative aspect.

**Table 5:** Reduplication encoding aspectual meaning in the elicited data.

Aspect type	N (analyzed)	Predicate reduplicated
Habitual	38	8 (21 %)
Continuative	33	8 (24 %)
Iterative	52	37 (71 %)
Total	123	53 (43.1 %)

As described in Section 3.2.2, it was analyzed statistically whether there is a difference between aspect types and between data sets in terms of reduplication. There was no significant difference between habitual and continuative aspect (*p* = 0.41); however, participants were five times more likely to use reduplication with iterative aspect than with habitual and continuative (odds ratio = 5.47, *p* < 0.001, *z* = 4.86, 95 percent confidence interval from 2.8 to 10.87). While I cannot conclude anything about the difference between habituals and continuatives in terms of likelihood of reduplication, iterative aspect is thus clearly more likely to be encoded by predicate reduplication than habitual and continuative aspect. Moreover, there was a significant effect of data set: participants were two times more likely to use reduplication in the corpus data than in the elicited data (odds ratio = 2.17, *p* = 0.03, *z* = 2.15, 95 percent confidence interval from 1.07 to 4.4). No significant interaction between data set and aspect type was found (*p* = 0.08 for continuative/habitual compared to iterative; *p* = 0.8 for continuative compared to habitual). An overview of all statistical data for the fixed effects is provided in Appendix B, and see van Boven (2023) for the complete analysis in R.

#### 4.1.1 Phonological restrictions on reduplication

Following Hoiting and Slobin (2001), it may be expected that predicates that are not reduplicated are body-anchored and/or involve internal movement. In total, 68 body-anchored verbs, 37 internal-movement verbs, and 67 verbs without constraining features were elicited. Recall, however, that not all elicited verbs could be analyzed for reduplication. Table 6 shows the numbers excluding those verbs that could not be analyzed for reduplication.

**Table 6:** Verb types in the elicited data; numbers after exclusion of predicates that could not be analyzed for reduplication.

Verb type	N (analyzed)
Body-anchored	56
Internal movement	18
No constraining features	49

Table 5 showed that out of the 123 elicited predicates that could be analyzed for reduplication, only 53 were in fact reduplicated. We can thus analyze whether the 70 non-reduplicated predicates involve internal movement or are body-anchored. However, this is not the case: out of the 70 non-reduplicated predicates, only 15 involve internal movement, and 26 are body-anchored. The other 29 verbs are not specified for one of the constraining features, suggesting that reduplication is optional even for verbs that are unconstrained; see Table 7.

**Table 7:** Potentially constraining features and reduplication in the elicited data; numbers after exclusion of predicates that could not be analyzed for reduplication.

Body-anchored/internal movement	Reduplicated	Not reduplicated
Yes	33	41
No	20	29

The Pearson Chi-Squared Test I conducted in R (R Core Team 2008), in order to investigate the relation between potentially constraining features (body-anchoredness/internal movement) and reduplication (i.e., Table 7), shows no significant relation between the two variables (*X* ^2^ (1, *N* = 123) = 0.05, *p* = 0.82). I cannot conclude anything as to whether there is a relation between the potentially constraining features on the one hand and predicate reduplication/zero marking on the other. An overview of all statistical data is provided in Appendix B, and see van Boven (2023) for the complete analysis in R.

We can, however, check for each aspect type whether predicates that involve internal movement or are body-anchored are reduplicated, and which ones these are. Table 8 shows the results.

**Table 8:** Reduplication of “constrained” predicates per aspect type in the elicited data (BA = body-anchored, IM = internal movement); shaded cells highlight unexpected patterns.

Verb type	N	Continuative	Habitual	Iterative
Reduplicated BA	30	4 (13 %)	5 (17 %)	21 (70 %)
Reduplicated IM	3	0 (0 %)	0 (0 %)	3 (75 %)

As Table 8 shows, the phonological restrictions previously identified for habitual and continuative aspect do not hold for iteratives in the elicited data: 24 predicates that are body-anchored or involve internal movement are reduplicated for this aspect type. For continuative and habitual aspect in the elicited data, the previously identified constraint on reduplicating internal-movement predicates seems to hold. However, for the phonological feature body-anchoredness, the picture is less clear, as there is a total of nine cases where a body-anchored predicate is reduplicated (the shaded cells in Table 8), namely hug (*n* = 3), sleep (*n* = 1), and melt (*n* = 5). Although at first glance, it thus seems that these contradict the phonological constraint previously identified, it is worth inspecting them further.

**Figure 4:**
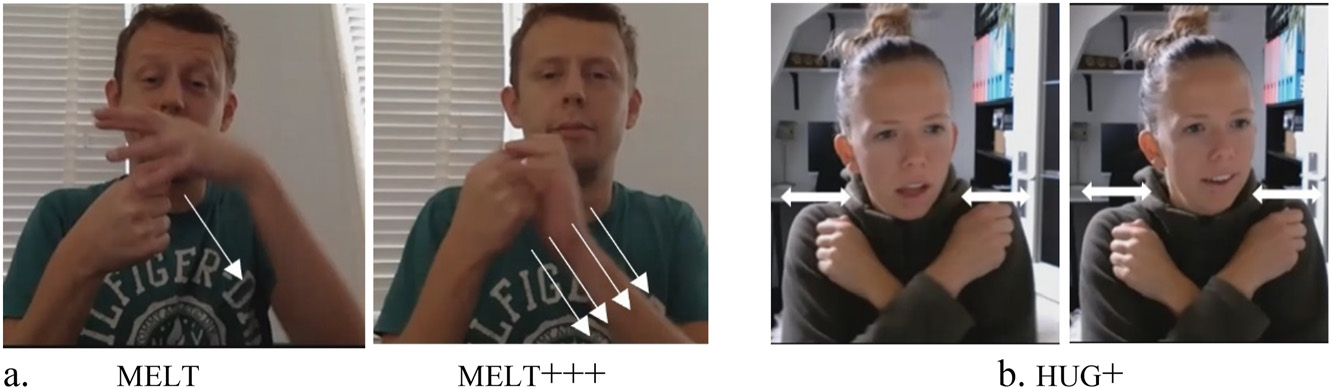
Reduplication of body-anchored melt (a) and hug (b).

The five instances of melt involve a variant of this verb that was analyzed as body-anchored, because the non-dominant hand is the place of articulation, while the dominant hand moves. Here, the verb actually does not contact the body, but rather the other hand – and in fact, the hands are often in proximity to each other, rather than actually making contact. This verb and its reduplicated form are illustrated in Figure 4a. For hug, it appears that it actually cannot be reduplicated in the expected way, that is, by repeating the entire path movement performed by the hands. Still, some participants employ an alternative strategy to reduplicate this body-anchored verb. The data suggest that hug is lexically specified for a short, swaying body movement. Some participants reduplicate the verb by repeating this body movement rather than the path movement of the hands. The hands thus retain contact with the body, as illustrated in Figure 4b. Finally, for sleep, it appears that the repetition may well have been a slip of the hand (but see an alternative explanation below: it contacts not the trunk, but the head).

**Figure 5:**
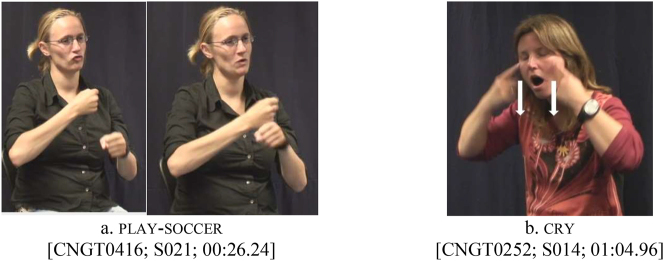
“Body-anchored” predicates play-soccer (a) and cry (b). For aspectual inflection, both can be reduplicated by repeating the path movement.

For the corpus data, it was more challenging to analyze potential effects of phonological features on reduplication, since it was obviously impossible to deliberately include base forms with different phonological specifications in this data set. Still, when we take a closer look at the corpus data in light of the patterns observed in the elicited data, some striking observations can be made. For both continuative and habitual contexts, it appears that body-anchored predicates can be reduplicated by repeating the path movement, but this only holds for a specific type of body-anchoredness in the corpus, namely predicates that contact the non-dominant hand, e.g., play-soccer in Figure 5a (much like melt in the elicited data), or the head, e.g., cry in Figure 5b (much like sleep in the elicited data).

**Figure 6:**
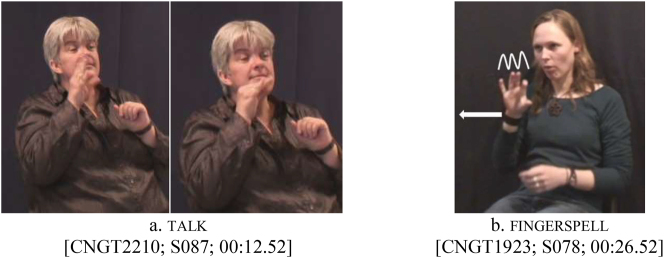
“Internal-movement” predicates talk (a) and fingerspell (b). For aspectual inflection, only fingerspell can be reduplicated by repeating the path movement.

As for predicates with internal movement in the corpus (e.g., handshape change in talk in Figure 6a), it appears that they are not reduplicated for habitual and continuative aspect, much like the elicited data. There is, however, one exception: wiggling of the fingers (as in fingerspell in Figure 6b and type) does not block aspectual reduplication.

Thus, the data suggest that, in the context of aspectual marking, both body-anchoredness and internal movement should be defined more narrowly – in fact, body-anchoredness could be re-defined as the location feature [trunk], while internal movement only comprises a change in finger position (or aperture, e.g., in the case of talk a change from open to closed). Moreover, as for predicates involving [trunk], it appears that only reduplication of *path movement* is blocked rather than all types of repetition (as evidenced by the repetition of body movements for hug in Figure 4b). These refinements should be taken with due caution, however, as our data do not involve negative evidence, and the data extracted from the corpus do not systematically include verbs for all phonological specifications. It remains to be investigated whether these constraints also hold outside of the current data set.

Finally, another important note should be added: the phonological restrictions identified in the data only apply when reduplication expresses specific types of aspect only. In some of the corpus sentences, reduplication seems to also mark multiple arguments (object and/or subject) on the verb, i.e., plurality, as in (16), and it is sometimes ambiguous whether reduplication actually encodes plurality of arguments, aspect, or both.

(16)

**Table:** 

[…] hearing **talk.2h.alt+ ** through-each-other theater palms.up
‘[…] All of the hearing people were (continuously) talking over each other during their play.’
[CNGT0294; S018; 02:09.81]

It seems that in cases in which reduplication (also) expresses plurality, verbs with an opening or closing of the hand can be reduplicated, as evidenced by the reduplication of talk in (16), which involves a handshape change – for this type of marking (but not for “pure” aspectual marking), the second hand is often added, and sometimes the hands move in alternation, as (16) also demonstrates. This type of reduplication occurred with internal-movement verbs where the hands open and close, like talk, observe, and accept. For corpus data, it is notoriously difficult to disentangle the functions that reduplication of the predicate may have in a specific sentence. This may also partly explain why participants were two times more likely to reduplicate the predicate in the corpus data than in the elicited data (see above), as such cases, in which reduplication may have multiple functions, have also been included in the corpus data, while other potential meanings of reduplication were controlled for in the elicited data (see also Section 5.3 for this and further possible explanations for the difference).

#### 4.1.2 Past and non-past

The phonological restrictions on reduplication identified in the previous section cannot be the sole reason why the predicate is not always reduplicated. As already mentioned for the elicited data, many of the non-reduplicated predicates are not specified for one of the potentially constraining phonological features and could thus, in principle, have been reduplicated, but are not. We now turn to another potential explanation as to why reduplication does not always occur: whether the sentence is situated in the past or not.

Events situated in both the past and the non-past were elicited (recall that NGT does not mark tense on the verb, but rather uses time adverbials). For the elicited sentences that could be analyzed for reduplication it was checked whether events situated in the past are marked more often than events in the non-past, following Oomen’s (2016) results. Table 9 shows the percentages for all aspect types together. These percentages do not suggest a clear difference between past and non-past in the data. The Pearson Chi-Squared Test I conducted in R (R Core Team 2008), in order to investigate the relation between past tense and reduplication (i.e., Table 9), shows no significant relation between the two variables (*X* ^2^ (1, *N* = 123) = 0.71, *p* = 0.4). I thus cannot conclude anything as to whether there is a relation between past/non-past contexts on the one hand, and predicate reduplication/zero marking on the other. An overview of all statistical data is provided in Appendix B, and see van Boven (2023) for the complete analysis in R.

**Table 9:** Reduplication of the predicate in past and non-past sentences for all aspect types in the elicited data.

	N (analyzed)	Predicate reduplicated	Predicate not reduplicated
**Past**	47	23 (49 %)	24 (51 %)
**Non-past**	76	30 (39 %)	46 (61 %)

Oomen (2016) only reported the difference between past and non-past for habituals, however. Table 10 shows the percentages of reduplicated and non-reduplicated predicates for habituals (that could be analyzed for reduplication) specifically, and the percentages are exactly the same for both tenses. In fact, they are in line with the more general frequency of reduplication in the elicited data (see Table 5). (17) illustrates the use of reduplicated predicates in a habitual past (17a) and non-past (17b) context.

**Table 10:** Reduplication of the predicate in past and non-past habitual sentences in the elicited data.

		N (analyzed)	Predicate reduplicated	Predicate not reduplicated
**Habitual**	**Past**	14	3 (21 %)	11 (79 %)
	**Non-past**	24	5 (21 %)	19 (79 %)

(17)

a.

**Table:** 

index_3a_ man index_3a_ live home. index_3a_ so-far every period+ after
lunch **clean+**
‘That man lives at home. Up until now, he has cleaned every day after lunch.’
[p01]

b.

**Table:** 

man person_3a_ index_3a_ every evening home **clean+**
‘That man cleans his home every evening.’
[p03]

Van Boven and Oomen (2021) already demonstrated that for the habituals in the corpus data, the vast majority of sentences situated in the past (i.e., 91 %) contains some type of manual marking; the fact that this percentage is higher than in the elicited data can be explained by two facts: (i) van Boven and Oomen subsumed under manual marking both reduplication and marking by means of an adverb, and (ii) reduplication occurs more frequently in the corpus in general – see Section 4.1.

More generally, given that I do not find any significant relation between past and non-past sentences on the one hand, and reduplication/zero marking on the other for the elicited data, I cannot conclude anything as to whether tense restricts aspectual reduplication in NGT (but see van Boven and Oomen [2021] for a discussion on the effect of datatype).

#### 4.1.3 Movement characteristics

**Figure 7:**
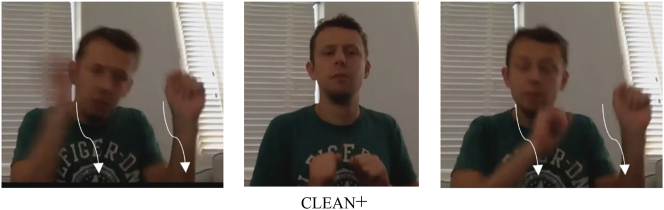
Iterative reduplication of clean with a pause in between reduplication cycles.

Cross-linguistically, different aspect types have been found to be distinguished from each other by differentiating the manner and rate of the movement of the reduplication cycles (e.g., Klima and Bellugi 1979; Rathmann 2005).14According to Hoiting and Slobin (2001), the modulation for habitual aspect in NGT is slower than for continuative aspect, and both involve an elliptical movement, while according to Oomen (2016: 43), the reduplication for continuative aspect is “relatively slow”, and elliptical modulation is observed for neither habituals nor continuatives in NGT. Here, I do not address differences in relative speed, as this feature was difficult to systematically analyze. In general, there did not appear to be clear differences in terms of speed, and an elliptical modulation also was not consistently observed in the data. In the elicited data, we observe one striking difference between iterative aspect on the one hand, and habitual and continuative aspect on the other: in the former, the movement cycles are often separated from each other by means of pauses, which is not the case for the latter two aspect types, where the movement cycles are uninterrupted. Iterative reduplication with pauses is illustrated in Figure 7.

**Figure 8:**
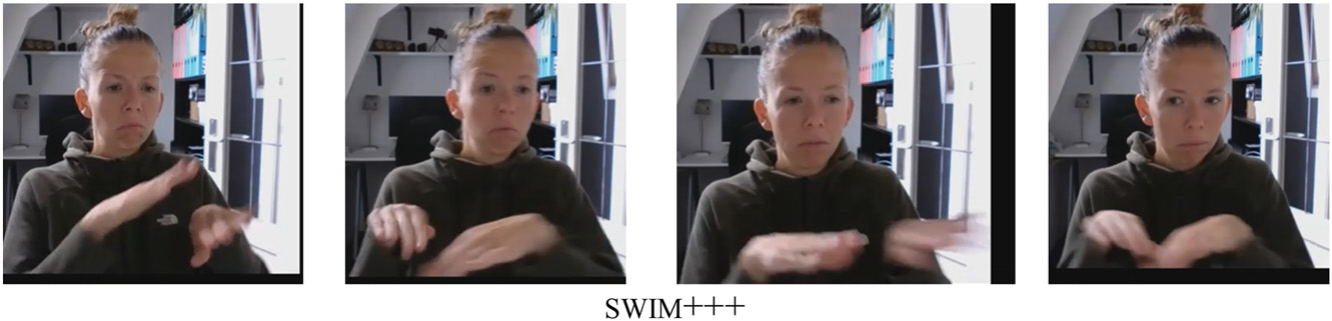
Iterative reduplication of swim with displacement of the reduplicants in space.

Finally, a characteristic of aspectual reduplication which had not been described in previous studies but which appeared in the data is spatial displacement of the predicate: sometimes, the reduplicants are articulated at different locations in the signing space. This is illustrated for swim in Figure 8, where there are four articulations of the verb, alternately on the left and right side of the signer.

### 4.2 Other aspectual markers in NGT

Apart from reduplication of the predicate, two other aspectual markers could be identified in the data: the sign glossed as cont (Section 4.2.1) and some non-manual markers (Section 4.2.2).

#### 4.2.1 Free aspectual marker cont

Remember that Hoiting and Slobin (2001) identified cont as a free aspectual marker, which, in their data, consistently follows body-anchored and internal-movement verbs and takes on the aspectual inflection. While Hoiting and Slobin describe this marker for both habitual and continuative aspect, in our data, it is only used to express continuity – likely an effect of the diverging definitions of habituality (cf. also van Boven and Oomen 2021).

In the elicited data, cont appears only 11 times (6 % of all elicited sentences). Most of these (*n* = 8) involve continuative contexts, as in (18a). Sometimes, cont occurs in a habitual (*n* = 2) or iterative (*n* = 1) sentence, but in all of these cases, it encodes the continuity of the event expressed in that sentence, as is true for the talking event in the habitual (18b).

(18)

a.

**Table:** 

man person_3a_ index_3a_ yesterday home. 4-hour long **cont+** clean+ […]
‘That man was home yesterday. He cleaned for four hours […]’
[p03]

b.

**Table:** 

index_3a_ child+ index_3a_ last week class out been. so-far every evening together **cont** talk […]
‘Those children went on a class outing last week. Every night, they talked continuously […]’
[p01]

Out of the 11 elicited sentences with cont, six could be analyzed for reduplication of the predicate. In three of those, both cont and the predicate are reduplicated (18a), while in the other three, only cont is reduplicated. As for the remaining five instances, cont is reduplicated in four of them. As for the potentially constraining factors body-anchoredness and internal movement, we observe that cont co-occurs both with constrained (*n* = 5) (18b) and unconstrained (*n* = 6) (18a) predicates – that is, if we apply the broader definition of “constrained”, thus including all types of body contact and internal movement. If we apply the narrower definition proposed in Section 4.1.1, cont combines with a constrained predicate in only four of the cases. Clearly, in the elicited data, cont does not (only) occur as a particle taking on the aspectual inflection in contexts in which the verb cannot be inflected.

In the corpus data, cont appears 26 times (11 % of all corpus sentences); it is mostly used as an aspect marker in continuative sentences (*n* = 21). It also occurs in habituals (*n* = 2; see van Boven and Oomen 2021) and iteratives (*n* = 3), but in those cases, again, it is not used as a marker of those aspect types; rather it expresses that the continuity of the action in that sentence. This use of cont to encode continuity in combination with another aspectual meaning was already identified in the corpus data analyzed by van Boven and Oomen (2021: 172).

#### 4.2.2 Non-manual markers

Both Hoiting and Slobin (2001) and Oomen (2016) identified several non-manual markers for habitual aspect (lax lips and protruding tongue, slowly circling head movement, left-to-right body movement) and continuative aspect (pursed lips and blowing gesture, back-and-forth body movement). The elicited data were analyzed for each of these markers, but none of them seems to express habituals/continuatives consistently in the data.

However, if we look at body and head movements and/or leans more generally in the elicited data, i.e., without differentiating between left/right and back/forth, 127 out of all 172 elicited sentences are non-manually marked in this way (74 %).15Potentially, head and body movement back-and-forth and left-and-right are different realizations of the same feature. Previous studies have described similar results, i.e., grammatical contexts where different instantiations of a certain non-manual, such as brow movement (raising vs. lowering), express the same meaning. See, for instance, Gökgöz (2011) on non-manual marking in Turkish Sign Language negation, and Klomp (2019) on non-manual marking in NGT conditionals. These include: 31 continuative sentences (67 % of all elicited continuatives), one of which has already been provided in (18a), and is repeated in (19a); 42 habitual sentences (67 % of all elicited habituals), one of which has been presented in (17a), and is repeated in (19b); and 54 iterative sentences (87 % of all elicited iteratives), exemplified in (19c).

**Table:** 

body back-and-forth

(19)

a.

**Table:** 

man person_3a_ index_3a_ yesterday home. 4-hour long cont + clean+ […]	
‘That man was home yesterday. He cleaned for four hours […]’	
[p03]	
body left-to-right

b.

**Table:** 

[…] index_3a_ so-far every period+ after lunch clean+	
‘[…] Up until now, he has cleaned every day after lunch.’	
[p01]	
body lean forward

c.

**Table:** 

man home index_3a_ . yesterday total four time clean. again one time	
clean++. four time wave.	
‘That man is home. Yesterday he cleaned four times. Still once again he cleaned. Four times!’	
[p07]

These examples also show that the scope of the non-manual marker may vary: it may accompany the predicate alone (19b), the manual markers (particles) and the predicate (19c), or may spread over the entire sentence (19a). It should be noted, however, that in reality, the numbers of occurrence are likely to be slightly lower, since in the data it is not always entirely clear whether a specific body or head movement indeed expresses aspect (e.g., in some cases, it might encode an enumeration rather than aspect). Still, these high percentages are rather striking.

For the corpus data, body and head movements are also the most prevalent non-manual markers, marking 35.9 % of the habituals (cf. van Boven and Oomen 2021), and even 76.4 % of the continuatives and 60.7 % of the iteratives – again, rather high percentages for the latter two, especially for corpus data.

## 5 Discussion

The results presented in the previous section show that reduplication can express habitual, continuative, and iterative aspect in the NGT data. Yet, while reduplication is a frequent concomitant of these three aspects, it does not obligatorily express aspect as a grammatical category. Why reduplication does not always occur, is not immediately clear – with respect to reduplication for all aspect types together in the elicited data, I cannot conclude anything about the relationship between past tense and reduplication, nor about the relationship between constraining phonological features and reduplication. Still, for habitual and continuative aspect, there appear to be rather specific phonological constraints on reduplication in the data, while this is not the case for iterative aspect. The continuative marker cont occurs, but not only with constrained predicates.

Section 5.1 further discusses the phonological constraints, while Section 5.2 addresses the finding that there is no formal difference between habituals and continuatives in the data, while iteratives are clearly distinct. This section also briefly addresses the optionality of aspectual reduplication I observe. The results presented here paint a picture that is rather different from previous research; both methodological and sociolinguistic factors could play a role, as discussed in Section 5.3.

### 5.1 Reconsidering phonological constraints on aspectual reduplication in NGT

The present study offers a new perspective on the phonological constraints on aspectual marking in NGT that have previously been put forward by Hoiting and Slobin (2001). Considering all aspect types in the elicited data together, no significant relationship was found between the phonological features of the predicates (i.e., internal movement/body-anchored or not) and reduplication, and thus I cannot conclude anything as to whether there is a relationship. Still, if we have a closer look at specifically the body-anchored and internal-movement predicates in imperfective contexts in our data, it seems that the constraints “body-anchored” and “internal movement” require a more precise definition, as proposed in Section 4.1.1. Independent evidence for the more specific definition of these constraints comes from phonological models developed for sign languages. First, for the “body-anchored” constraint, I argued that in the current data predicates articulated on the trunk are phonologically constrained, while predicates contacting other body parts are not. In phonological models of sign languages – e.g., Sandler’s (1989) Hand Tier model and Brentari’s (1998) Prosodic model – it is assumed that signs are specified for a major location, the trunk or torso being one of them, as opposed to, for instance, the non-dominant hand and the head. The fact that these major locations are distinguished in these models supports the proposal that one major location, the trunk, blocks reduplication, while the others do not.

Second, for internal movement, I noted that, in our data, handshape change blocks reduplication, but finger wiggling does not. In the phonological models mentioned above, finger wiggling and handshape change are represented differently, despite the fact that both are hand-internal movements. Recall that finger wiggling has been subsumed under trilled movement, and it is represented as such in both models. Sandler (1993: 252) defines trilled movement as “rapidly repeated hand-internal movement”, and in her Hand Tier model, signs with trilled movement have a feature [trill]. In Brentari’s (1998: 164) Prosodic model, trilled movement is a so-called “articulator-free feature”. Handshape change, however, is not represented by specific features in the phonological models. In the Hand Tier model, a change in finger position is represented by branching at the finger position node (see, e.g., Sandler 1993; Sandler and Lillo-Martin 2006). In the Prosodic model, one underlying handshape is specified, while the other (redundant) handshape can be predicted from the opposing value of the underlying shape at the aperture node (Brentari 1998). The fact that finger position/aperture changes on the one hand, and trilled movement on the other, are represented separately in both models is in line with the observation that only one of them blocks reduplication. To further investigate this, it would be interesting to test whether other types of trilled movement (such as circling, rubbing, and hooking, as identified by Brentari) also block reduplication in NGT.

Based on these observations, I propose to revise the previously identified phonological constraints on aspectual reduplication in NGT (Hoiting and Slobin 2001), as in (20).

(20)

**Table:** 

*Proposed revised constraints on aspectual reduplication in NGT (version 1)*

a.

**Table:** 

In the habitual and continuative aspect, the major location feature [trunk] blocks reduplication of verbs.

b.

**Table:** 

In the habitual and continuative aspect, handshape change (i.e., a change in finger position/aperture) blocks reduplication of verbs.

Interestingly, these constraints do not apply to iteratives in the data set. Indeed, as mentioned in Section 4.1, participants were five times more likely to use reduplication with iterative aspect than with habitual and continuative aspect, which may result from this difference in terms of constraints on reduplication. Possibly, combining a change in finger position with movement repetition is too complex when the movement cycles are uninterrupted, as is true for habituals and continuatives. In iteratives, the reduplication cycles are separated from each other by means of pauses, making the movement combinations less complex. As for the [trunk] location, it is less evident why a combination with repeated movement would yield a phonologically too complex form (in fact, there are verbs in NGT which are lexically specified for both body-anchoredness and repeated movement, such as be-scared, which contacts the trunk). Still, the resistance of body-anchored signs to undergo reduplication appears to be a more general phenomenon: for plural reduplication, van Boven (2021) found that NGT nouns specified for inherent repetition or body contact are less likely to be reduplicated than nouns without those features, although reduplication was not completely blocked. In other sign languages, body-anchoredness has been observed to completely block plural reduplication (Pfau and Steinbach [2005] for German Sign Language; Pizzuto and Corazza [1996] for Italian Sign Language; Sutton-Spence and Woll [1999] for BSL).

The fact that iterative reduplication is never blocked in the data suggests that there is an interaction between the phonological make-up of a sign and the aspect type: the phonological constraints are specific to the inflectional morpheme. An interaction between the phonology, morphology, and reduplication type has also been described by Harley and Leyva (2009) for Hiaki, an Uto-Aztecan language, although the findings do not exactly correspond to what is found here for NGT. They report five different reduplication types, and the application of two of the reduplicative allomorphs (the disyllabic and closed-syllable reduplication) is determined by a combination of not only phonological, but also morphological (transitivity-marking suffixes) properties of the stem.

Yet, it is not immediately clear which morphological distinction we are dealing with in NGT: iterative versus habitual versus continuative, or perfective versus imperfective more generally. This matter is the focus of the next section.

### 5.2 Reconsidering grammaticalized aspectual distinctions in NGT

The data in this study were collected from a semantic starting point, that is, the sentences were divided into habitual, continuative, and iterative aspect based on their meaning, and only after that, their form was analyzed. In doing so, I identified potential phonological constraints on habitual and continuative reduplication, but not on iterative reduplication, where the movement cycles are separated by means of pauses. Additional evidence for the distinct status of iteratives comes from another striking feature: the sign again can sometimes intervene between different instances of the verb, as in (21).

(21)

**Table:** 

hug again hug again hug again hug
‘They hugged several times / they hugged again and again.’
[p01]

Here, the additional material appears in between single instances of the verb, i.e., instances with one movement cycle. Two potential explanations for the insertion of again can be offered. It is possible that the pauses in iterative reduplication allow for the insertion of (phonologically light) material in between reduplication cycles. Or, alternatively, the insertion of again in between several instances of the verb actually came first, and is in the process of grammaticalization into the iterative reduplication with pauses between reduplicants. The data are uninformative as to which explanation is the correct one, leaving this question open for future analyses. For the other two reduplication types, this type of inter-cycle insertion is never observed in the data.

The fact that iterative reduplication is optional in both data sets, despite not being phonologically constrained, suggests that it is not (yet) completely grammaticalized (pointing towards the second explanation offered in the previous paragraph). Indeed, more generally, there is quite some variation, since reduplication alternates with sentences without reduplication for all three aspects. This is reminiscent of Johnston et al.’s (2015) study on what they call finish-type signs in Auslan, which express perfective aspectual meaning: they suggest that the grammaticalization of such signs “is not well advanced” (p. 152) (see Johnston et al. [2015] for more elaborate discussion). The present data suggest the same for NGT aspectual reduplication.

Other studies, again in line with the present one, also describe optionality and variation in sign language aspectual marking. Recall from Footnote 6 that aspectual reduplication (denoting repeated or continuing events) was found to be optional in Auslan (see Gray 2013),16Recall also from Footnote 6 that for Gray (2013) this optionality is one of the reasons to reject a morphological analysis. However, here, unlike Gray, I do observe that fixed reduplication types express fixed aspectual meaning in NGT, which is in line with previous studies suggesting aspectual morphemes, which have fixed forms. and Palfreyman (2019) describes variation in the expression of completion in the urban sign language varieties of Solo and Makassar (Indonesia). Such variation and optionality is not entirely unexpected, as it has also been described for other grammatical domains in NGT (e.g., Oomen and Pfau [2017] for variation in negation; van Boven et al. [2023] for the optionality of negative concord), as well as in other sign languages (e.g., Fenlon et al. [2018] for optionality of verb modification in BSL, and the factors conditioning it; Palfreyman [2019] for variation in negation in the Solo and Makassar varieties).

When there is reduplication, however, iteratives are clearly distinct from habituals and continuatives in the data. Recall from Section 2.2.1 that earlier research on other sign languages already showed that aspect types can be distinguished by movement modulations such as adding pauses in between reduplication cycles (e.g., Klima and Bellugi 1979). The fact that NGT iterative reduplication is distinguished from other aspects by means of this modulation is thus not surprising from an intra-modal perspective. Interestingly, several spoken languages have also been described to employ different types of reduplication for different aspect types. For instance, in Coos (a now extinct isolate from Oregon), reduplication of the first syllable expresses “intensity of action, repetition, duration, and customary action” (Frachtenberg 1922: 377), while reduplication of the final syllable expresses “distribution, mutuality, and, in intransitive verbs, an action that is performed now and then” (Frachtenberg 1922: 380). The reduplication types thus have several different functions, some of them aspectual. Mithun (1999) presents similar findings on the Salish language family, where most languages are described to have three reduplication types that have different (some aspectual) functions. Similarly, in Hiaki, different reduplicant shapes are semantically contrastive, but only for specific verbs (e.g., *noka* ‘speak’ and *vahume* ‘swim’). A light-syllable reduplicant is used to derive habitual meaning for these verbs, while a reduplication + gemination form expresses emphatic, idiosyncratic, or iterative meaning (Harley and Leyva 2009). Although this is not described as a general pattern across all verbs in Hiaki, the parallel with the NGT data (and data from other sign languages) in using different types of reduplication to express iterative or habitual meaning is notable.

While the iteratives I analyzed thus appear to be distinct from the habituals and continuatives in terms of their reduplicative form, a formal distinction between the latter two could not be established in this study. In fact, in the present data, they are expressed by the same type of reduplication, without pauses in between, which appears phonologically constrained. I take this to suggest that the semantic distinction between habitual and continuative may not be grammaticalized in NGT in the form of verbal inflection. Recall from Section 2.1 that the habitual and continuous aspects have been proposed as a subdivision of imperfective aspect more generally (Comrie 1976), while iterative aspect is perfective. It thus might be that, when there is reduplication, NGT distinguishes perfective and imperfective, but does not make a further formal distinction between habitual and continuous. If this is indeed the case, then the phonological constraints in (20) can be revised again, as in (22).

(22)

**Table:** 

*Proposed revised constraints on aspectual reduplication in NGT (version 2)*

a.

**Table:** 

In the imperfective aspect, the major location feature [trunk] blocks reduplication of verbs.

b.

**Table:** 

In the imperfective aspect, handshape change (i.e., a change in finger position/aperture) blocks reduplication of verbs.

From a cross-modal perspective, this finding is not surprising. As described in Section 2.1, Bybee (1985) reports that most spoken languages in her sample distinguish only the imperfective and the perfective inflectionally (see also Dahl and Velupillai 2013b). This pattern was actually more common than further distinguishing habitual and continuous. Compare (23), from Spanish, where the imperfect can express habitual or continuative meaning,17Spanish does have a separate progressive form, but this is optional, as the imperfect does not exclude a progressive reading (Comrie 1976). to (24ab), where NGT also uses one and the same form to express habitual (24a) and continuative (24b) meaning.

(23)

**Table:** 

*Juan llegaba.*
‘John was arriving.’
‘John used to arrive.’
(Spanish; Comrie 1976: 25)

(24)

a.

**Table:** 

man index_3a_ home index_3a_ . index_3a_ each evening **clean+**.
‘That man is at home. He cleans every evening.’
[p01]

b.

**Table:** 

man home. yesterday four-hour long **clean+**.
‘That man is at home. Yesterday, he was cleaning for four hours.’
[p04]

From an intra-modal perspective, however, the findings presented here are more striking. In the literature on aspect marking in sign languages, specific verbal modulations for habituals or continuatives are often mentioned, as we also saw in Section 2.2.1 (e.g., Cabeza Pereiro and Fernández Soneira [2004] for Spanish Sign Language; Rathmann [2005] for ASL; Sutton-Spence and Woll [1999] for BSL). The fact that the NGT data exhibit one form for both habituals and continuatives, without modulating the movement, situates NGT differently from other sign languages in the landscape of aspectual inflection, thus adding to our understanding of intra-modal variation in this domain.

So far, it has been assumed that habitual and continuative aspect are both instances of the imperfective viewpoint. Note, however, that Rathmann (2005) analyzes these as instances of situation aspect in ASL.18
Rathmann (2005) argues that ASL has only two viewpoint morphemes: one to mark perfective viewpoint (the particle finish), and one to mark imperfective viewpoint (the conative morpheme). The other morphemes (including habitual and continuative) are argued to contribute situation-type aspect, as they can co-occur with the perfective marker, and they are concerned with duration, telicity, and dynamism (which is characteristic of situation aspect, according to Rathmann); see also Footnote 5. While our data are not informative as to whether the continuative/habitual morpheme(s) could be analyzed as an instance of situation-type aspect, the NGT data are different from ASL in at least one way: we cannot formally distinguish habituals from continuatives. To put it differently: the semantic distinction between habitual and continuous does not appear to be grammaticalized based on the NGT data – irrespective of whether they are situation- or viewpoint-type. For now, I assume that they are instances of imperfective viewpoint, and leave this matter for future studies. Such studies could focus on the question whether the reduplication that is used to encode both continuatives and habituals can combine with a perfective marker in NGT. If we are in fact dealing with imperfective viewpoint, the expectation would be that this combination is ruled out.

This outcome stands in sharp contrast with findings reported in previous studies on NGT: while Hoiting and Slobin (2001) and Oomen (2016) did not agree on the specific form of the marking, they both identified a formal distinction between habituals and continuatives. A likely explanation lies in the different methods of the studies: the previous studies on NGT only considered elicited data from a limited number of participants. The same is true for several previous studies on other sign languages (e.g., Cabeza Pereiro and Fernández Soneira [2004] consider only two participants for LSE). In contrast, this study draws on corpus and elicited data and includes more signers, and consequently, the data more closely represent the language as it is actually used. Further methodological considerations, as well as potential sociolinguistic variation, will be discussed in the next section.

### 5.3 Methodological and sociolinguistic considerations

The combination of corpus data and elicited data has several important implications that should be taken into account. First, there are some, mostly quantitative, differences between the data sets. For instance, while iteratives are relatively rare in the corpus (*n* = 28), they are the most frequent aspect type in the elicited data (*n* = 62). Footnote 13 already indicated that, while there is a significant relation between the data set (corpus or elicited) and aspect type (habitual, continuative, or iterative), this result might, in fact, be due to several dependencies in the data, most likely the fact that I targeted an equal amount of sentences for each aspect type in the elicited data, which may have resulted in an unusually high number of iteratives as compared to the naturalistic language use in the corpus data. In addition to this, the fewest searches were performed for iteratives on the translation tier (see Table 1), indicating that Dutch might have fewer particles expressing iteratives than particles expressing the other two aspect types. Iteratives are thus difficult to find in the corpus because they might be less likely to be overtly marked in Dutch.19Or one could speculate that (overtly marked) iteratives are generally infrequent in natural language use, which might be an alternative explanation as to why phonological constraints have not (yet) developed for this aspect type.


Another difference between the two data sets lies in the frequency of predicate reduplication, as the statistical analysis showed that corpus participants were two times more likely to reduplicate the predicate than participants in the data elicitation (see Section 4.1). Again, there are a few possible explanations. In the elicited data, phonological features of the predicates were controlled for, and the stimuli thus included a fair number of predicates that cannot be reduplicated. Obviously, in the corpus data, it was impossible to control for such phonological features. Another potential explanation is that in the corpus data, different functions of reduplication were sometimes difficult to disentangle. This challenge has already been illustrated in Section 4.1.1 with (16), repeated here as (25), where it is ambiguous whether the reduplication expresses plurality of arguments, aspect, or both. It is thus quite possible that I sometimes annotated reduplication of the predicate as encoding aspectual meaning, when it actually was not used as such, resulting in an overestimation of how often the predicate was reduplicated for aspect. In the elicited data, this likely did not happen, as the potential meanings of reduplication could be controlled for (e.g., it was ensured that none of the sentences had a distributive meaning).

(25)

**Table:** 

[…] hearing **talk.2h.alt+ ** through-each-other theater palms.up
‘[…] All of the hearing people were (continuously) talking over each other during their play.’
[CNGT0294; S018; 02:09.81]

Finally, the difference in frequency of reduplication between the two data sets might also be due to how the data sets were compiled. I collected the corpus sample for the largest part by searching on the translation tier, selecting sentences with aspectual meaning. This way of collecting the sample did in principle not influence the type of aspect marking I would find: aspectual meaning could be expressed in several ways – e.g., by particles, reduplication, or both. This is different for the elicited data, given that there were aspectual particles in the stimuli in order to make clear which aspectual meaning was targeted. Recall, for example, the stimulus in Figure 2, which translates as “What has he been doing for hours?”. Participants, answering the stimulus questions in full sentences, often repeated this overt aspectual marker, in this case, “for hours”. It could be the case that such lexical indicators removed the need to also reduplicate the predicate. At the same time, it is also clear that lexical indicators and reduplication are not completely in complementary distribution: for instance, in the corpus data, reduplication and adverbs co-occur in 26.4 % of the habituals (see van Boven and Oomen 2021), in 27.4 % of the continuatives (these numbers might be higher if only unconstrained predicates are included in the data), and in 21.4 % of the iteratives. Also, if they were completely complementary, one would expect no reduplication at all in the elicited data – in fact, double marking is not uncommon in sign languages (e.g., in several sign languages, there is double marking of agreement, by inflection of the main verb and an auxiliary; see Steinbach and Pfau [2007]). Still, it is expected that the elicited data do not reflect the frequency of aspectual reduplication in natural language use, while the corpus data likely do.

Second, there is some variation between signers in our data. For instance, in the elicited data, one signer is overrepresented in reduplicating body-anchored signs for continuative/habitual aspect, namely the predicates melt, which involves contact with the non-dominant hand (see Figure 4a), and hug, a body-anchored sign (see Figure 4b), for which he repeats the body movement rather than the path movement: four, i.e., half, of the instances come from this signer. On the other hand, there is one signer in the elicited data who never reduplicates body-anchored and internal-movement predicates for habitual or continuative aspect. This suggests that it may, to some extent, be signer-dependent how strictly the phonological constraints are interpreted. Potentially, sociolinguistic factors play a role here. Interestingly, the signer who strictly maintains the phonological constraints is from Groningen, and Hoiting and Slobin (2001), who identified these constraints, based their observations on informants from the same region. The participant who is overrepresented in the reduplication of melt and hug, on the other hand, is from Amsterdam, and Oomen (2016), who did not find any restrictions on reduplication, tested a participant from this region. This is further evidence for regional variation in aspectual marking – something that Oomen already suggested as an explanation for the diverging findings. This should be researched further, by more systematically including signers from different regions.20As mentioned previously, regional variation in NGT is normally assumed to be limited to the lexicon (Schermer 2004). However, aspectual marking is not the only grammatical domain in NGT where potential regional variation has been observed: van Boven et al. (2023) suggest that there may be regional variation in the use of Negative Concord in this language.


Because of the nature of the data, I cannot answer all open questions. A first drawback is that the data do not provide negative evidence. In order to further test phonological constraints on aspectual reduplication, grammaticality judgment tasks should be conducted. Moreover, the data suggest that reduplication is not obligatory, even for non-restricted predicates: the elicited data included 29 non-restricted predicates that were not reduplicated for habitual or continuative aspect (see Section 4.1.1). Iteratives appear not to be subject to phonological constraints, and still 29 % of the elicited iteratives does not involve reduplication of the predicate. The corpus data also show inter-signer variation in the use of reduplication: some signers occasionally use reduplication to mark aspect on unconstrained predicates, but not always. We could thus conclude that aspectual reduplication is not obligatory, but it still remains unclear whether it is truly optional, or whether other (potentially syntactic or prosodic) factors are at play here. I consider this an avenue for further research.

Finally, as mentioned in Section 3.3.2, the statistical analyses reported here have some drawbacks. I reported the results of a logistic regression model, but the estimates should be taken with due caution, given the small sample size (even though the corpus and elicited data were taken together) (see Moineddin et al. 2007). Further, given that the elicited data constitutes an even smaller sample, for this data set, I reported chi-squared analyses, which assume that the observations are independent of each other, while this is not the case in the current data set (e.g., multiple observations per participant). It is hoped that in future research, the outcomes reported here can be investigated further based on a larger sample, in order to conduct logistic regression models and report the (more reliable) estimates. However, given the (relatively small) population under study, this may be a challenging undertaking.

## 6 Conclusions

This study investigated reduplication of the predicate to encode habitual, continuative, and iterative aspect in NGT, taking into account both corpus and elicited data. The results show that reduplication can express all three aspect types, i.e., it is a frequent concomitant of habitual, continuative, and iterative aspect, but is not obligatory. Moreover, it appears to be phonologically constrained for habitual and continuative aspect: verbs which have [trunk] as their major location and verbs which involve a handshape change were never reduplicated in the data. For iterative aspect, no phonological constraints can be identified, and I hypothesized that this is a consequence of the fact that iterative reduplication cycles are separated by means of pauses, which is not the case for habituals and continuatives. There are no formal differences between the latter two in the data, which suggests that NGT may mark imperfective aspect more generally. If this is indeed the case, this would be a surprising finding given previous research on NGT and other sign languages, but in line with what has been reported for many spoken languages (e.g., Bybee 1985).

Additional questions remain for further research, some of which I address here. First, it would be interesting to conduct grammaticality judgments to test (i) whether continuative/habitual reduplication can co-occur with a perfective particle, and (ii) whether verbs with trilled movement other than wiggling can be reduplicated. This could provide more insight into whether continuative/habitual reduplication indeed encodes the imperfective viewpoint more generally, and would allow us to test whether handshape change (i.e., finger position/aperture) is indeed the only type of internal movement that blocks reduplication. Second, future studies should thoroughly explore whether synchronic variation reflects grammaticalization of NGT aspect marking and corresponding phonological constraints (cf. Johnston et al. [2015] on Auslan) – such studies could also uncover linguistic and/or contextual factors underlying the variation (for instance, whether there is a difference in aspect marking between monologues/narratives and dialogues, a factor which was found to be relevant in the analyses of aspect marking by Johnston et al. [2015] on Auslan and by Palfreyman [2019] on the urban sign language varieties of Solo and Makassar in Indonesia).

Finally, a comparison of some of our findings to those reported in previous studies on NGT (Hoiting and Slobin 2001; Oomen 2016) suggests that regional variation might be at play in the domain of aspectual marking; more specifically, it appears that signers from Groningen adhere to the phonological constraints more strictly than signers from Amsterdam (as also suggested by Oomen [2016]). It would be interesting to further investigate potential sociolinguistic factors that influence NGT aspect marking, by systematically including signers from different regions, but also of different ages and genders (cf. Johnston et al. 2015; Palfreyman 2019).

## Appendix A: Glossing conventions for the sign language examples

sign: The gloss of one single sign
sign-sign: Multiple words form the gloss of one single sign
sign++: Reduplication of a sign; number of pluses indicates number of repetitions
index_x_: Pointing sign with a linguistic function (pronoun); subscript number refers to locations in the signing space (1 = chest of signer; 3a/3b = right or left in signing space)
poss_1_: Sign indicating possession by first person
sign.2h.alt: One-handed base sign articulated by two hands moving alternately
palms.up: Multifunctional, mostly clause-final particle
xxx: Non-manual marker; line indicates the scope
[CNGTx; Sx; x:x]: Corpus NGT file number; signer number; begin time (m:s.ms)
[p0x]: Participant number in elicited data

## Appendix B: Overview of results from the statistical analyses

See Table B1, Model 1 and 2, Table B2, Model 3, Table B3, Model 4.

**Table B1:** Expected and observed values for aspect types in the corpus and elicited data.

Aspect type	Corpus		Elicited	
	Expected	Observed	Expected	Observed
Habitual	98.45	106	70.55	63
Continuative	89.13	106	63.87	47
Iterative	52.43	28	37.57	62
Total	240		172	

**Model 1:** Pearson’s Chi-squared test for aspect type (habitual, continuative, and iterative) and corpus/elicited data (Table B1). Significant findings (*p* ≤ *0.05*) are indicated by an asterisk (*).

*X* ^2^	*Df*	*N*	*p*
36.303	2	412	<0.001*

**Model 2:** predicate.reduplication ∼ Aspect.type * Data.set + (Aspect.type | Participant.number). Significant findings (*p* ≤ *0.05*) are indicated by an asterisk (*).

Predictor	Estimate	Odds ratio	95 % CI	*z*	*p*
Aspect type (−conthab+it)	1.70	5.47	2.76–10.87	4.86	<0.001*
Aspect type (−cont+hab)	−0.28	0.75	0.38–1.48	−0.82	0.41
Data set (−elicited+corpus)	0.78	2.17	1.07–4.4	2.15	0.03*
Aspect type (−conthab+it) : Data set (−elicited+corpus)	−1.15	0.32	0.09–1.15	−1.75	0.08
Aspect type (−cont+hab) : Data set (−elicited+corpus)	−0.17	0.84	0.22–3.25	−0.25	0.80

**Table B2:** Expected and observed values for reduplication/zero marking of potentially constrained and unconstrained verbs in the elicited data.

Phonological features	Reduplication of verb		No reduplication of verb	
	Expected	Observed	Expected	Observed
Potentially constrained	31.89	33	42.11	41
Unconstrained	21.11	20	27.89	29
Total	53		70	

**Model 3:** Pearson’s Chi-squared test with Yates’ continuity correction for reduplication/zero marking and constrained/unconstrained verbs in the elicited data (Table B2). Significant findings (*p* ≤ *0.05*) are indicated by an asterisk (*).

*X* ^2^	*Df*	*N*	*p*
0.05	1	123	0.82

**Table B3:** Expected and observed values for reduplication/zero marking in past and non-past contexts in the elicited data.

Tense	Reduplication of verb		No reduplication of verb	
	Expected	Observed	Expected	Observed
Past	20.25	23	26.75	24
Non-past	32.75	30	43.25	46
Total	53		70	

**Model 4:** Pearson’s Chi-squared test with Yates’ continuity correction for reduplication/zero marking and past/non-past contexts in the elicited data (Table B3). Significant findings (*p* ≤ *0.05*) are indicated by an asterisk (*).

*X* ^2^	*Df*	*N*	*p*
0.71	1	123	0.4
